# Atopic dermatitis: pathogenesis and therapeutic intervention

**DOI:** 10.1002/mco2.70029

**Published:** 2024-12-08

**Authors:** Chengcheng Yue, Hong Zhou, Xiaoyan Wang, Jiadong Yu, Yawen Hu, Pei Zhou, Fulei Zhao, Fanlian Zeng, Guolin Li, Ya Li, Yuting Feng, Xiaochi Sun, Shishi Huang, Mingxiang He, Wenling Wu, Nongyu Huang, Jiong Li

**Affiliations:** ^1^ State Key Laboratory of Biotherapy and Cancer Center West China Hospital Sichuan University Sichuan University and Collaborative Innovation Center for Biotherapy Chengdu Sichuan China; ^2^ Department of Cardiology West China Hospital Sichuan University Chengdu Sichuan China

**Keywords:** atopic dermatitis, immune cells, pathophysiology, therapeutic intervention

## Abstract

The skin serves as the first protective barrier for nonspecific immunity and encompasses a vast network of skin‐associated immune cells. Atopic dermatitis (AD) is a prevalent inflammatory skin disease that affects individuals of all ages and races, with a complex pathogenesis intricately linked to genetic, environmental factors, skin barrier dysfunction as well as immune dysfunction. Individuals diagnosed with AD frequently exhibit genetic predispositions, characterized by mutations that impact the structural integrity of the skin barrier. This barrier dysfunction leads to the release of alarmins, activating the type 2 immune pathway and recruiting various immune cells to the skin, where they coordinate cutaneous immune responses. In this review, we summarize experimental models of AD and provide an overview of its pathogenesis and the therapeutic interventions. We focus on elucidating the intricate interplay between the immune system of the skin and the complex regulatory mechanisms, as well as commonly used treatments for AD, aiming to systematically understand the cellular and molecular crosstalk in AD‐affected skin. Our overarching objective is to provide novel insights and inform potential clinical interventions to reduce the incidence and impact of AD.

## Graphical abstract

Atopic dermatitis (AD) is a chronic pruritic inflammatory skin disease, and its complex pathogenesis is closely linked to genetic and environmental factors, skin barrier dysfunction as well as immune dysfunction, which imposes a significant socioeconomic burden in all countries. The compromised function of the skin barrier leads to the release of alarm substances and triggers activation of the type 2 immune pathway, resulting in recruitment various immune cells to the skin for coordinated cutaneous immune responses. In this review, we comprehensively outline the methodologies of experimental AD models and extensively discuss therapeutic interventions, with a focus on the contributions of various skin immune cells to the regulatory mechanisms of AD. The precise characterization of the functional plasticity within these skin immune cells will facilitate more accurate targeting of specific functional modules, thereby offering broad therapeutic applications for the prevention of AD.

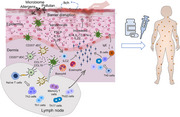

## 1 INTRODUCTION

Atopic dermatitis (AD) is the most common inflammatory skin disorder worldwide, affecting 15–20% of children and 10% of adults.^1^, ^2^ Notably, AD ranks as the leading skin disease in children worldwide.^3^ The Global Burden of Disease study identified AD as the 15th most impactful nonfatal disease and the leading skin disorder globally, highlighting its substantial burden on populations worldwide.^1^, ^4^ As the largest organ in the human body, the skin functions as a robust physicochemical, microbiological, and immune barrier, providing multifaceted protection.^5^ As the primary immunoprotective barrier for nonspecific immunity, it houses an intricate network of resident immune cells and is composed of the epidermis, dermis, and subcutaneous layers.^6^, ^7^ The epidermis is differentiated from the ectodermal layer and is a complex layer of squamous epithelial cells that can be divided into the stratum corneum (SC) and tight junctions (TJs).^8^, ^9^ This intricate arrangement provides the skin with its permeability barrier function. However, in AD, these protective structures are compromised, making the skin more susceptible to environmental factors, triggering type 2‐dominated inflammation and perpetuating a destructive cycle, further compromising the integrity of the SC structure.^10^ While, the dermis consists of collagen, elastic fibers, and fibroblasts, along with various resident immune cells and in response to inflammatory stimuli by rapidly recruiting additional immune cells.^11^, ^12^, ^13^ The interplay between skin barrier dysfunction and disruption of immune homeostasis is central to the pathogenesis of inflammatory skin diseases like AD.^12^, ^14^, ^15^

AD typically begins in childhood and often persists into adulthood, with the appearance and extent of lesions varying according to age, race, and ethnicity.^16^, ^17^, ^18^ The onset of AD frequently coincide with allergen sensitization, initiating a sequence known as the “atopic march,” in which AD can advance to other atopic conditions, including asthma and allergic rhinitis.^19^ Beyond physical symptoms, individuals with AD commonly experience comorbidities such as depression, anxiety, and sleep disturbances, which collectively diminish overall well‐being.^20^ The pathophysiology of AD is complex and is strongly influenced by genetic susceptibility.^21^ AD is generally classified as a type 2 inflammatory disorder, characterized by a complex interaction between epidermal barrier defects and immune dysregulation.^22^ Clinically, AD presents with acute pruritus, erythematous papules with scaling, plasma exudation, and itching, which significantly impair the epidermal barrier, leading to dysregulation of immune‐modulating proteins, including interleukins (ILs) IL‐25 and IL‐33, in skin epithelial cells.^18^, ^23^, ^24^, ^25^, ^26^ These proteins initiate and promote Th2 cell‐mediated immune responses and release cytokines such as IL‐4 and IL‐5, which then stimulate B cells to produce IgE.^27^, ^28^, ^29^ Furthermore, elevated levels of histamine and total IgE in the peripheral blood of individuals with AD promote the accumulation of eosinophils and mast cells (MCs) in AD‐lesions, thereby exacerbating the release of inflammatory mediators and intensifying symptoms.^30^, ^31^, ^32^, ^33^

Based on the 2019 findings of the Global Burden of Disease Alliance, approximately 171 million individuals, or roughly 2.23% of the global population, are significantly affected by AD.^34^ Consequently, investigating the pathogenesis of AD remains a critical area of research. AD is characterized by a compromised skin barrier and is driven by complex interactions among genetic, environmental, and microbial factors, as well as immune dysregulation, which contribute to its development.

In this review, we offer an overview of both foundational knowledge and recent advances in understanding the pathophysiology of AD, with a particular emphasis on the role of immune cells and immune dysregulation. We summarize the methodologies used in experimental AD models and extensively discuss therapeutic interventions. Our aim is to present a comprehensive overview of the latest insights into the multifaceted mechanisms underlying AD pathology, while also highlighting state‐of‐the‐art therapeutic approaches.

## 2 PATHOGENESIS OF AD

AD is a chronic inflammatory disease characterized by a multifactorial etiology and complex pathogenesis.^35^ Its development has been linked to various factors, including genes such as abnormalities in immune and skin barrier genes, environmental influences, and microbial dysbiosis, particularly colonization by *Staphylococcus aureus*. One primary pathophysiological theory suggests that dysfunction of the natural skin barrier is central to AD's development, with both genetic and environmental factors, as well as microbial dysbiosis, affecting its proper functioning.^36^ Additionally, AD is a persistent inflammatory skin condition that is closely associated with immune system dysregulation. Disturbances in both innate and adaptive immune responses, including cytokine release, are believed to play a pivotal role in the pathogenesis of the disease. A comprehensive understanding of AD pathogenesis is crucial for effective drug development. This section begins by summarizing the contributions of each factor, with a particular focus on the pathogenic role of immune dysregulation in AD.

### 2.1 Genetic factors

Genetic factors play an important role in the development of AD. Family history is a key risk factor for AD, with heritability estimated to range from 80 to 90% in twin studies, underscoring the significant contribution of genetic background to disease susceptibility.^37^ To date, genome‐wide association studies have identified 91 specific genomic regions linked to susceptibility for AD.^38^ The filaggrin gene (FLG) is the most well‐established genetic factor associated with AD, and loss‐of‐function variants in FLG strongly linked to an increased risk of AD, predisposing individuals across diverse demographic groups worldwide.^39^, ^40^, ^41^, ^42^, ^43^ In addition to FLG, multiple other genetic susceptibility loci such as Trichohyalin and serpin family B member 7, of have been identified that may affect the development of AD.^44^, ^45^, ^46^ Furthermore, a study by David and colleagues identified that residues associated with AD susceptibility were linked to the G polymorphism of SNP rs9277534 in the 3′ UTR of the HLA‐DPB1 gene, suggesting a higher expression of these HLA‐DP alleles. Conversely, protective residues were associated with the A polymorphism, indicating a lower expression.^47^ Moreover, rare protein‐coding variants account for 12.56% of AD heritability, with novel genome‐wide significant susceptibility genes, such as Docking protein 2 and CD200 receptor 1, identified as contributing factors.^48^ Additionally, mutations in immunomodulatory genes, including IL‐4, IL‐13, and IL‐6R, which influence immune dysregulation, also play a significant role in the pathogenesis of AD.^49^, ^50^

### 2.2 Environmental and microbiome

While genetics play a crucial role, the rise in the prevalence of AD over the past few decades also points to the significant influence of environmental factors.^51^ Environmental changes, such as extreme temperature, heavy rains and floods, air pollution, and wildfires can affect and damage the epithelial barrier of the skin and respiratory tract, leading to damage of the epithelial cells and the release of alarmins, which activate the immune response and exacerbate AD.^52^ In recent years, it has been found that ecological dysregulation of the skin microbiome, which is regulated by genetic, environmental, and other factors, leads to skin barrier damage and promotes the development of AD.^53^ A growing number of research studies have reported that AD severity is directly proportional to absolute and relative abundance of *S. aureus* and appears to be partially related to host factors such as race, gender, and age.^54^, ^55^, ^56^ Moreover, specific strains and genomic loci of *S. aureus* and *Staphylococcus epidermidis* have been linked to AD status and geographical location.^57^ Additionally, accumulating evidence suggests that gut dysbiosis, particularly during infancy, is strongly associated with the development of AD, and it also been demonstrated that the gut microbiota, along with the production of toxic compounds, can influence and exacerbate AD symptoms through the gut–skin axis, involving immune, metabolic, and neuroendocrine pathways.^56^

### 2.3 Skin barrier dysfunction

**FIGURE 1:**
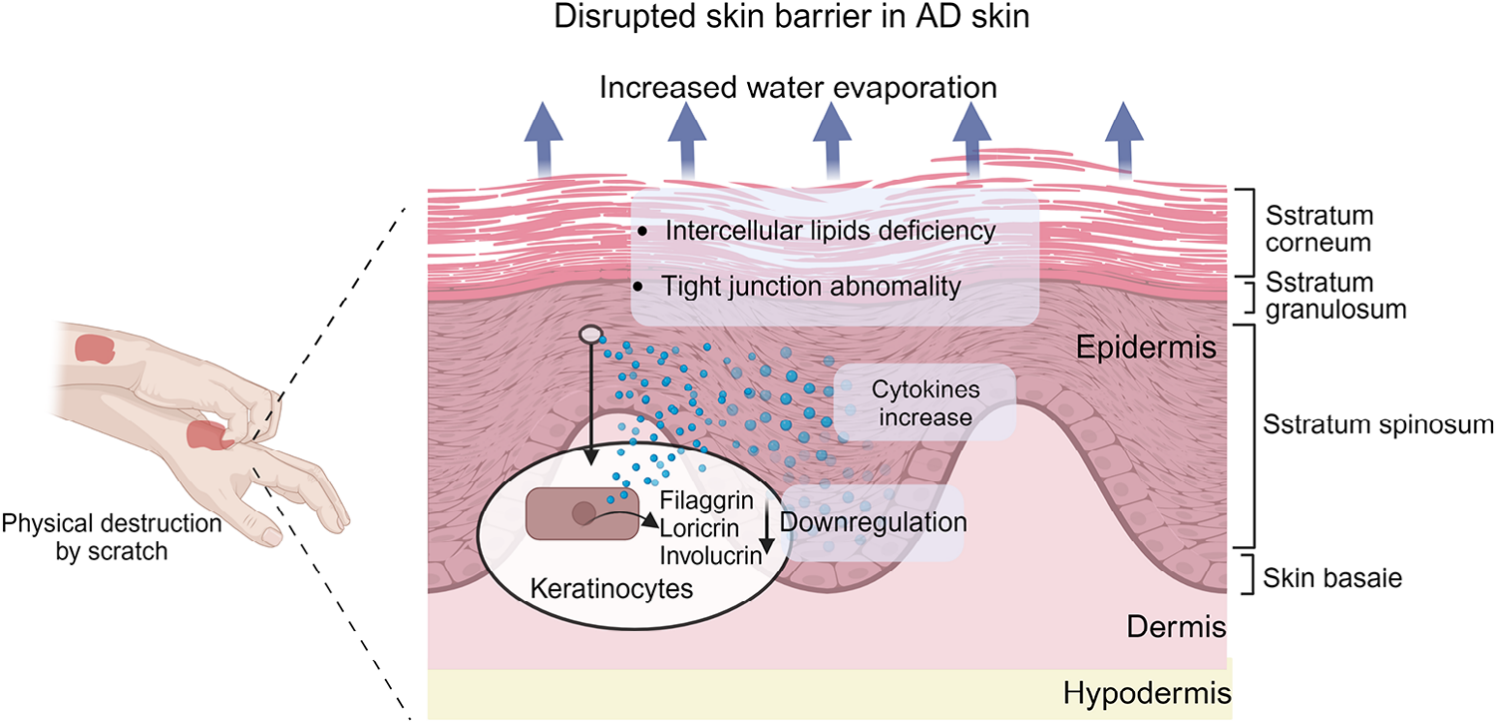
Skin barrier dysfunction and the role of keratinocytes in AD. The skin is composed of the epidermis, dermis, and subcutaneous tissue, with the epidermis itself subdivided into four distinct layers. Keratinocytes, the predominant cell type within the epidermis, play a crucial role in maintaining skin barrier integrity. In atopic dermatitis (AD), the absence of key genes such as Filaggin (FLG) in keratinocytes diminishes the expression of tight junction proteins. This resulting in compromised barrier function and disruption alters the localization of tight junctions within the granular layer. Concurrently, keratinocytes secrete inflammatory cytokines and chemokines, which exacerbate the inflammatory response characteristic of AD. The associated pruritus in patients further damages the skin barrier, contributing to increased transepidermal water loss. This figure was drawn by BioRender.com.

The compromised barrier function is a distinctive characteristic of AD and demonstrates a positive correlation with the severity of the condition.^10^ The causes of skin barrier dysfunction in AD are multifactorial, involving genetic mutations, activation of immune pathways, interactions between epithelial cells, and altered host defense mechanisms.^58^ Skin barrier damage promotes transepidermal water loss and penetration of allergens and microorganisms into the skin, leading to irritation, itching, and scratching, resulting in mechanical damage to the epidermis, which further exacerbates skin barrier damage.^59^, ^60^, ^61^ The barrier function of the skin is carried out mainly by the SC, the outermost layer of the epidermis.^15^ The SC is composed of a layer of keratinocytes (KCs) (corneocytes) embedded in a surrounding lipid matrix.^62^, ^63^ The integrity of the SC depends on the terminal differentiation of epidermal KCs, during which they mature into protein‐rich corneocytes enveloped by extracellular lamellae containing specific epidermal lipids.^58^ This intricate structure is essential for the skin's permeability barrier function.^64^ Lipid deficiency is a key feature of AD, and studies have demonstrated that abnormal expression of cholesterol, nonessential free fatty acids, and ceramides in the SC disrupt the lipid matrix that fills the SC, thereby damaging the skin barrier^63^, ^65^, ^66^, ^67^ (Figure 1). Previous studies of barrier dysfunction in AD have primarily focused on KC lipids. However, recent research has revealed that AD patients also exhibit an altered lipidome in sebum and abnormal lipid metabolism in sebaceous glands, and the amount of sebum secreted by sebaceous glands is reduced in patients with AD, which is negatively correlated with barrier function and disease severity.^68^

### 2.4 Immune cells dysregulation

**FIGURE 2:**
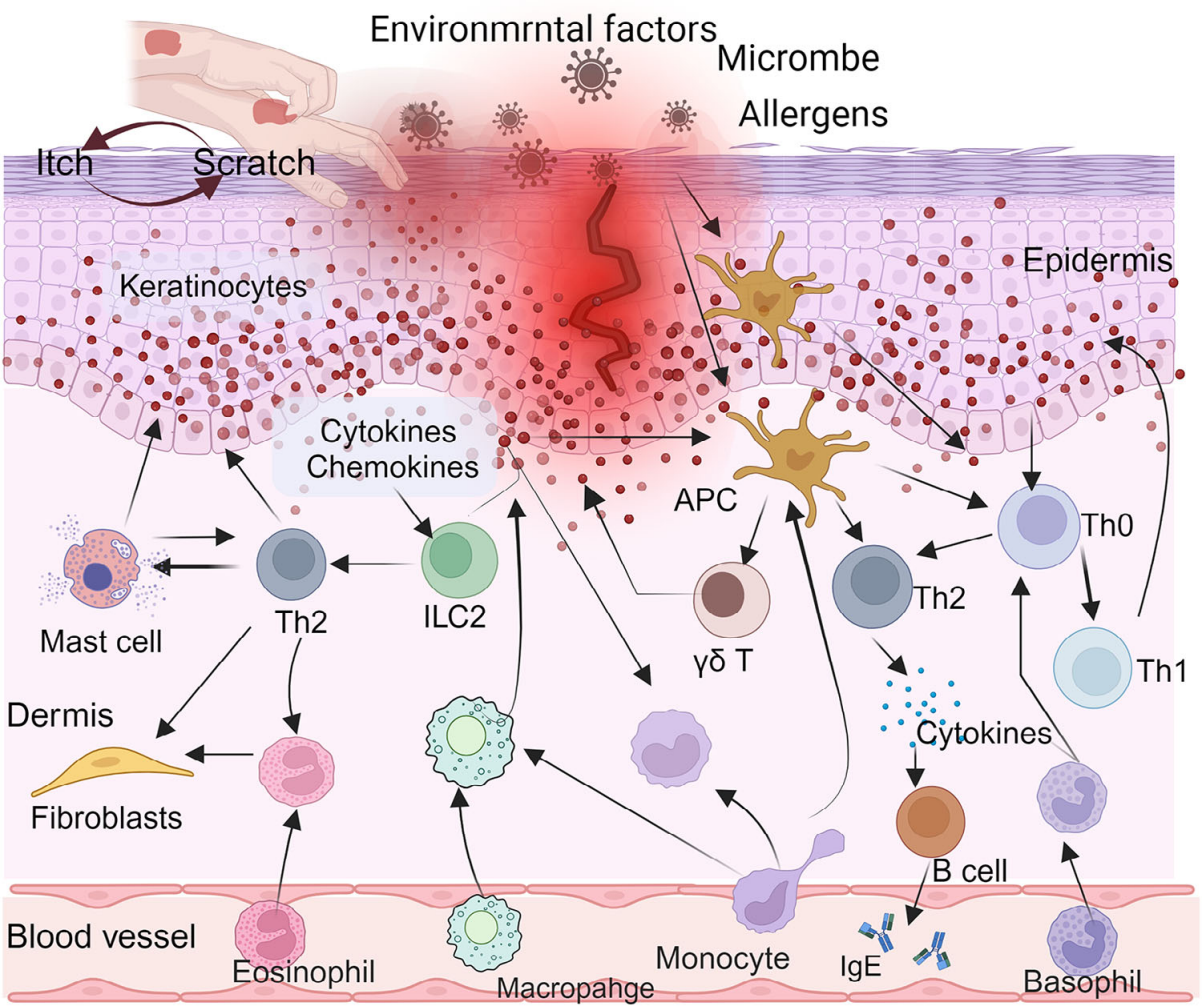
Immunological imbalances and cellular interactions in AD. Immune dysregulation, particularly T cell‐mediated responses, play a pivotal role in the pathogenesis of AD. A compromised skin barrier increases susceptibility to environmental allergens, triggering the activation of antigen‐presenting cells that stimulate T cells and initiate a cascade of inflammatory responses. Additionally, keratinocytes contribute to this process by secreting inflammatory mediators that promote immune cell infiltration into AD‐lesions, further exacerbating inflammation. The interactions among keratinocytes, fibroblasts, innate immune cells such as type 2 innate lymphoid cells, mast cells, basophils, eosinophils, monocytes, and macrophages, as well as adaptive immune cells (T cells and B cells) play a pivotal role in the pathogenesis and progression of AD. This figure was drawn by BioRender.com.

Skin barrier disruption can activate the immune response in a proinflammatory cascade. In AD, skin inflammation is caused by host–environment interactions involving KCs as well as many tissue‐resident immune cells, which produce cytokines.^69^ In the following section, we summarize the effects of immunological imbalances and cellular interactions in AD, focusing on the current roles of KCs, skin immune cells, and other cell types in the disease's pathogenesis (Figure 2).

#### 2.4.1 KCs in AD

KCs are the most abundant cell type in the epidermis,^6^ as the main component of the epidermal barrier, which secrete a variety of cytokines and chemokines that regulate the pathogenesis of various inflammatory diseases, including AD and psoriasis.^70^, ^71^ Epidermal KCs help maintain skin barrier function by controlling skin permeability and water evaporation through a network of structural proteins.^72^ KCs participate in the intracellular signal transduction cascade through pathogen recognition receptor (PRRs), such as Toll‐like receptors (TLRs) and Dectin‐1, thereby upregulating proinflammatory factors and chemokines, which subsequently leads to inflammatory cell infiltration, immune dysregulation, and ultimately promotes the pathophysiological process of AD.^73^, ^74^ Moreover, inhibiting KC production of alarmins or inflammatory factors can effectively prevent the development and progression of chronic inflammatory diseases like AD and psoriasis.^75^, ^76^ Therefore, KCs are considered a viable therapeutic target for interrupting the vicious cycle of chronic inflammation and alleviating symptoms associated with AD. Barrier dysfunction plays a pivotal role in the pathogenesis of AD. Diminished or absent expression of barrier‐associated proteins, such as filaggrin, loricrin, involucrin, and claudin, in KCs results in compromised skin barrier integrity, which contributes to the onset and progression of AD (Figure 1).^68^ Abnormal KC proliferation and differentiation are also significant in AD pathogenesis. A recent single‐cell analysis of skin biopsies from AD patients revealed an increased presence of IL19^+^IGFL1^+^KCs in chronic lesions, which may be involved in the disruption of the skin barrier.^77^ Activation of endogenous transient receptor potential vanilloid 3 (TRPV3) activity in KCs has been implicated in promoting the development of AD, but according to Yujing Wang's team, direct inhibition of TRPV3 with Scutellarein can ameliorate carvacrol‐induced proliferative and proinflammatory responses.^78^ Moreover, elevated expression of cytokines, such as IL‐25, secreted by KCs, has been observed in the skin of AD patients and is thought to contribute to the development of AD.^61^ Abnormal RNA splicing in KCs has also been linked to inflammatory skin diseases. A study by Xinhui Ni's team revealed that KCs with specific deletion of RNA deconjugating enzyme DDX5 were more prone to skin inflammation including AD, and that the cytokine IL‐17D could regulate skin inflammation such as AD by suppressing DDX5 expression in KCs through activation of the CD93–p38 MAPK–AKT–SMAD2/3 signaling pathway.^79^ In addition, Gupta's research team^71^ found that the absence of tumor necrosis factor (TNF) superfamily member 14 (known as LIGHT) or its receptor lymphotoxin beta receptor (LTβR), herpesvirus entry mediator (HVEM), in KCs protected mice from allergen‐induced skin inflammation such as AD. Furthermore, KCs produce various antimicrobial peptides capable of combating pathogenic bacteria and fungi, thereby influencing the development of AD.^73^

#### 2.4.2 Skin dendritic cells

**FIGURE 3:**
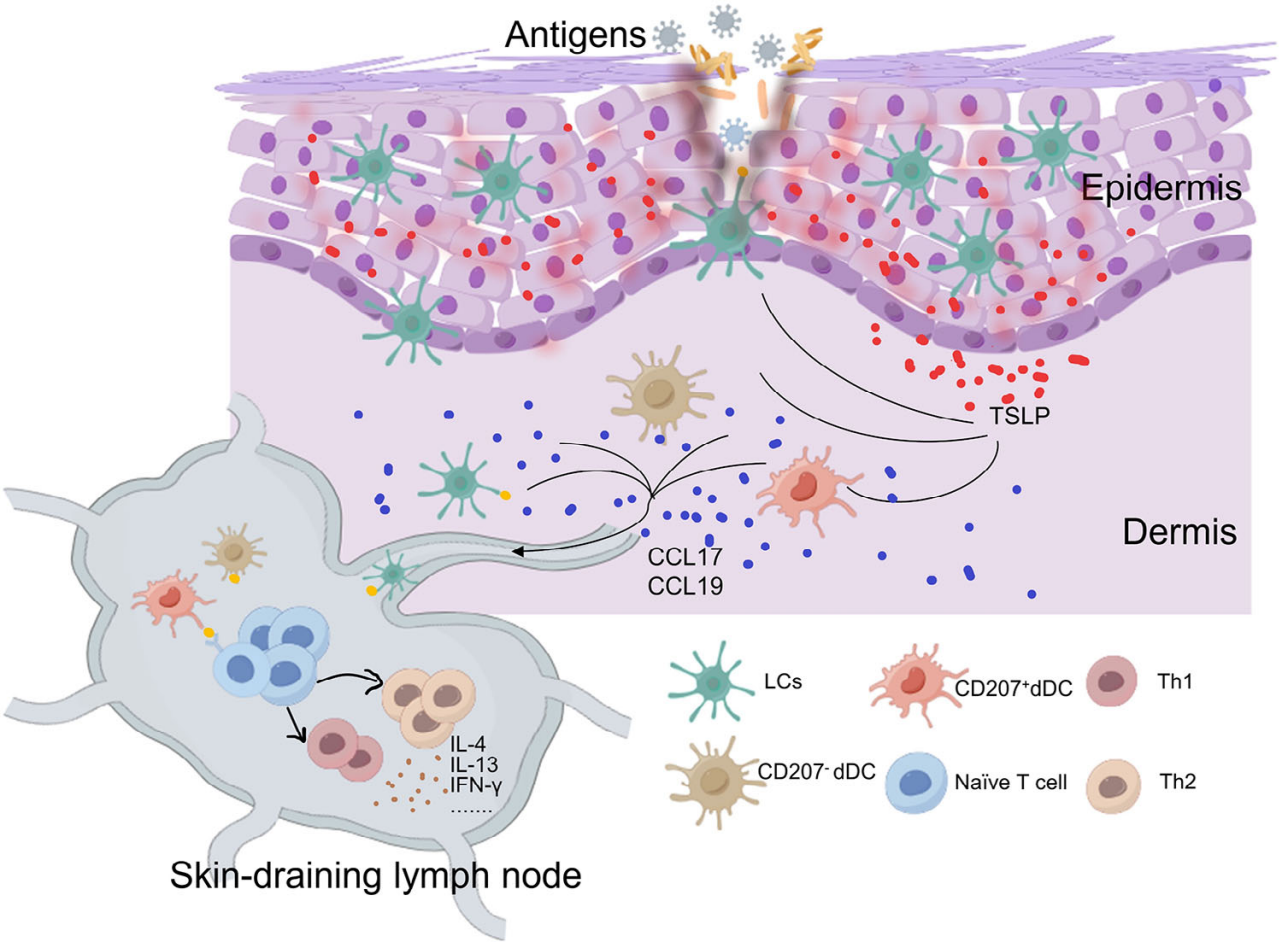
Participation of skin dendritic cells in pathogenesis of AD. In response to external stimuli, both LCs and dDCs migrate to the draining LN, but CD207^+^dDC migrate faster than LCs. LCs and dDCs are crucial for delivering antigens to CD4^+^T cells and promoting Th2 differentiation which in turn regulates the development of AD. TSLP, thymic stromal lymphopoietin; CCL17, chemokine ligand 17; CCL19, chemokine ligand 19. This figure was drawn by Figdraw.

Skin dendritic cells, the immune system's sentinels, exhibit cutting‐edge advances by producing immune responses to invading pathogens through the epidermis, playing a crucial role as professional antigen‐presenting cells, and their control extends to CD4^+^ T cells, affecting both homeostasis and inflammation.^80^, ^81^, ^82^ It is well known that DCs play a pivotal role in the immunologic cascade, prompting the AD initiation and development.^83^ Skin DCs are primarily divided into two subpopulations: Langerhans cells (LCs), situated in the upper basal layer of the epidermis, and dermal dendritic cells (dDCs), located in the connective tissue of the dermis.^84^ In response to extrinsic stimuli, skin dendritic cells are able to respond rapidly by migrating into the skin‐draining lymph nodes and activating other immune cells. The occurrence and development of AD are inextricably linked to DCs, and this section summarizes the recent research progress on LCs and dermal DCs in AD (Figure 3).

##### Langerhans cells

LCs are bone marrow‐derived dendritic antigen‐presenting cells that reside in the epidermis and other epithelia, serving as the principal antigen‐presenting cells of the epidermis.^85^, ^86^ LCs are integral to skin homeostasis by initiating innate and adaptive immunity in response to external stimuli and transporting antigens to antigen‐specific T cells in the skin‐draining lymph nodes.^87^, ^88^ The SC and TJs form a physical barrier in the epidermis, and the dendrites of activated LCs extend beyond TJs to capture external antigens.^89^, ^90^ Notably, the number of LCs penetrating TJs increases approximately fivefold in erythematous lesions of AD patients, but not in nonlesional skin, indicating a potential role of LCs in AD pathogenesis.^89^ Additionally, in the presence of thymic stromal lymphopoietin (TSLP) overexpression, LCs promote TSLP‐induced follicular helper T (TFH)/Th2 differentiation and further contribute to the progression of AD.^91^, ^92^ Skin microbiome homeostasis was dysregulated in AD patients, and AD severity was positively correlated with high *S. aureus* colonization.^93^, ^94^ In *S. aureus* colonized AD skin, both LCs and inflammatory dendritic cells (IDECs) showed immaturity, lacked high spontaneous migratory activity, and remained unresponsive to TLR2 ligands. The inhibition of TLR2‐mediated *S. aureus*‐associated signaling hindered *S. aureus* clearance, further promoting AD development.^95^, ^96^, ^97^ LCs interact directly with *S. aureus* through the PRR langerin (CD207), and the langerin protein interacts with *S. aureus* through the conserved β‐N‐acetylglucosamine modification of wall folic acid, which distinguishes *S. aureus* from other staphylococcal species and triggers a proinflammatory response similar to that observed in AD patients.^98^, ^99^ Mutations in the gene encoding the epidermal structural protein filamentous polyprotein (FLG) stand out as the most influential risk factor for AD development.^100^ A recent study suggest that individuals with FLG‐null mutations exhibit increased LC maturation markers in nonlesional skin, regardless of AD presence, linking FLG‐null mutations to enhanced LC maturity.^101^ Angeli's team^102^ found that administering BW245C, an agonist of prostaglandin receptor 1 (DP1), during the induction of AD in mice with ovalbumin (OVA) inhibited LC migration and Th2 cell production, which in turn inhibited the development of AD‐like skin lesions, suggesting that PGD2‐DP signaling in the LCs may be involved in one of the regulatory pathways in AD. After suppressing MyD88 in KCs, the induction of AD through OVA sensitization was observed to impede the migration of LCs and significantly hinder the development of AD‐like dermatitis. Additionally, the skin migration assay revealed that the application of contact allergens such as 1‐fluoro‐2,4‐dinitrobenzene (DNFB) or mite allergens on the ear skin also resulted in inhibited migration of LCs in MyD88.^103^ Since AD is a heterogeneous and highly complex disease, it is possible to further suffer from complications and develop other diseases, such as Eczema herpeticum (EH).^104^ The expression and activity of indoleamine 2,3‐dioxygenase in LCs are implicated in the pathophysiology of herpetic eczema in AD patients, potentially serving as biomarkers for predicting the risk of AD progressing to EH or other viral comorbidities, as reported by Staudacher and team.^105^ Furthermore, human LCs highly express CD1a molecules, which can interact with skin flora and *staphylococcal* phosphatidylglycerol antigens to activate T cells via CD1a, potentially influencing immune responses in *Staphylococcus*‐associated skin diseases.^106^, ^107^

##### Dermal dendritic cells

Similar to LCs, dDCs migrate to lymph nodes, functioning as adept antigen‐presenting cells proficient in initiating adaptive immune responses.^108^, ^109^, ^110^, ^111^ dDCs can be classified into different subsets based on the presence of many surface markers. The primary classification criterion hinges on the expression of Langerin (CD207), categorizing cells into Langerin^+^dDC and Langerin^−^dDC, with the latter constituting the predominant subset.^84^, ^112^ Further subtypes emerge, including CD207^+^CD103^−^dDCs, CD207^+^CD103^+^dDCs, CD207^−^CD11b^+^dDCs, and CD207^−^CD11b^−^dDCs, delineated by the expression of CD103 or CD11b.^84^, ^113^, ^114^ Langerin^+^dDCs can migrate to the skin and lymph nodes both under normal conditions and during inflammation. Unlike LCs, Langerin^+^dDCs achieve faster transit to the lymph nodes and undergo examination within 18 h of stimulation, enabling efficient presentation of peripherally acquired antigens to T cells.^115^ Recent studies have shown that elevated levels of TSLP in localized atopic lesions can stimulate the generation of bone marrow‐derived inflammatory Langerin^+^dDCs by inducing an upregulation in Langerin expression in AD mouse.^116^ Bedoui's research^108^ indicated that all dDC subpopulations could present antigens to CD4^+^ T cells; however, only the CD207^+^CD103^+^ dDC subpopulation efficiently presented herpes simplex virus type 1 (HSV‐1) antigens to naïve CD8^+^ T cells, highlighting their role in MHC class I‐restricted cross‐presentation.^108^ Lee et al.^117^ constructed AD models using repetitive protein patches to sensitize the epidermis in C57BL/6 or BALB/c mice, and the results showed that Langerin^−^dDC was still present and exhibited a Th2 response after intraperitoneal injection of DT in muLangerin‐DTR mice, suggesting that dDCs are critical for single‐epidermal sensitization of the two mouse strains to regulate the development of AD. In addition, there was a slight increase in the presence of BDCA3^+^DCs, known for their tolerogenic properties and ability to facilitate regulatory T‐cell differentiation, as well as BDCA2^+^plasmacytoid DCs, in lesional AD patients compared to controls.^118^ Although current research has demonstrated the regulatory role of dDCs in AD progression, further investigations studies are needed to clarify how dDCs modulate AD, delineate the specific contributions of distinct subgroups of dDCs in regulating AD, and unravel the underlying mechanisms involved.

#### 2.4.3 T cells

##### Th cells

**FIGURE 4:**
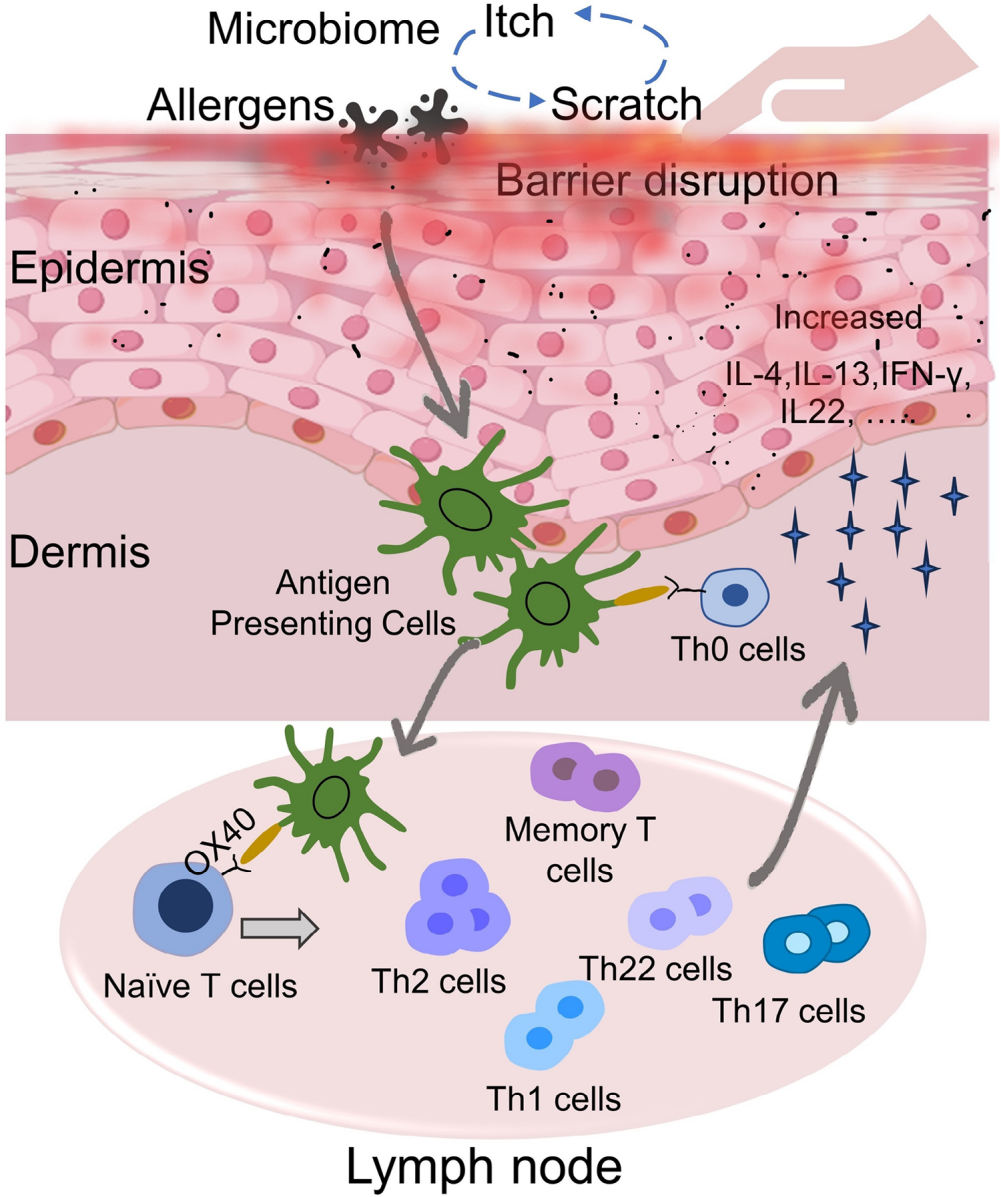
Th cells in pathogenesis of AD. Skin‐tropic effector T cell clones originate from naïve T cell precursors. Following skin barrier damage, various microbial invasions and antigen‐presenting cells activate T cells, triggering a cascade of T cell responses. These activated cells migrate to sites of inflammation, releasing inflammatory cytokines that exacerbate the progression of AD.

From an immunological perspective, AD is predominantly influenced by the Th2 pathway (IL‐4 and IL‐13), while also involving varying degrees of involvement from the Th1 (interferon [IFN]‐γ), Th22 (IL‐22), and Th17 (IL‐17 and IL‐26) immune pathways.^119^, ^120^ The cellular infiltration in AD lesions is largely composed of T lymphocytes, with distinct subsets of CD4+ helper T cells playing a significant role in the disease's progression. AD skin is characterized by a type 2 immune response, with Th2/Tc2 cells being major contributors to various pathological aspects of AD and serving as the primary source of elevated type 2 cytokines, such as IL‐4, IL‐5, IL‐13, and IL‐31.^76^ The presence of these cytokines leads to the apoptosis of KCs, inflammation associated with eczema, and itching, thereby confirming the involvement of T cells in the development of AD.^121^ Bangert's research team,^119^ through multi‐omics analysis with single‐cell RNA sequencing and multiplex proteomics, found that LAMP3^+^CCL22^+^ mature dendritic cells, CRTH2^+^CD161^+^T helper (T_H_2A) cells, and CRTAM^+^ cytotoxic T cells, which express high levels of CCL17 and IL13. Furthermore, T_H_2A cells demonstrated characteristic cytokine receptor patterns, expressing IL17RB, IL1RL1 (ST2), and CRLF2. These findings suggest that these cell types are pivotal responders to epidermal alerting proteins IL‐25, IL‐33, and TSLP, respectively, in AD.^119^ A recent study conducted by Jie Han's research team revealed that knockdown of the mouse transmembrane protein 232 (TMEM232) gene resulted in reduced infiltration of Th1 and Th2 cells, among others, in the skin lesion areas of MC903‐induced AD mice, effectively suppressing AD‐like lesions.^122^ C‐C motif chemokine receptor 4 (CCR4) serves as the primary chemokine receptor for Th2 and Th17 cells, and studies have indicated that inhibiting CCR4 may result in an antiallergic effect by impeding the recruitment and proliferation of Th2 and Th17 cells in AD.^123^, ^124^, ^125^ A Phase I clinical trial evaluating the CCR4 antagonist RPT193 in AD showed significant clinical improvement and modulation of the cutaneous transcriptomic profile in this inflammatory skin condition.^126^ Additionally, OX40 is expressed across various functional T cell subsets, with increased expression observed on cutaneous CD4^+^ T cells.^127^ The OX40/OX40L axis plays a crucial role in Th2‐mediated inflammation in AD, and the inhibition of OX40 signaling demonstrates potential in preventing the progression of AD. Currently, ongoing clinical trials are investigating its therapeutic efficacy^128^, ^129^, ^130^ (Figure 4).

##### Other T cells

γδ T cells have garnered research interest over the past few decades due to their critical role in allergic diseases.^131^ Impaired skin barrier function is a prominent characteristic of AD.^132^, ^133^ The absence of γδ T cells in mice was found to induce spontaneous AD, highlighting their potential as key regulators in skin barrier maintenance and promoting healing in AD‐related skin lesions.^134^, ^135^ Moreover, children with AD exhibit a notable increase in Vγ9Vδ2 lymphocytes.^136^ There is also an observed rise in systemic γδ T cells in AD children infected with *Staphylococcus aureu* *s*, further implicating γδ T cells in pathogenesis of AD.^137^ However, the precise mechanisms underlying this association remain to be investigated.

Additionally, a single‐cell transcriptome analysis of the skin in lesion areas of AD patients found that lesional AD samples were characterized by an expansion of and tissue‐resident memory (TRM) T cells (CD69^+^CD103^+^), as well as a higher prevalence of type 2 (IL13^+^)/type 22 (IL22^+^) T cells compared with type 1 (IFNG^+^) cells in lesional AD skin.^118^ Circulating skin‐homing cutaneous lymphocyte‐associated antigen (CLA)^+^T cells represent a subset of human memory T cells, with the majority of T cells in the skin expressing CLA. The observed positive correlation between the phenotype and quantity of circulating CLA^+^T cells and the severity of AD, along with the significant infiltration of CLA^+^T cells in AD‐affected skin, implies a potential role for circulating CLA^+^T cells as a peripheral cellular biomarker for AD.^138^ Zheng's team discovered that CD4^+^ TRM cells in initiating early responses in AD relapse and in driving chronic relapsing inflammation through neutrophil recruitment.^139^ Targeting CD4^+^ TRM cells could be a promising strategy for addressing chronic relapsing inflammation in AD. Pathogenic TRM cells, recently described subpopulation of CD69^+^CD103^+^ lymphocytes, have been shown to mediate a variety of chronic inflammatory diseases in multiple tissue types, and in the skin, TRM cells are known as resident skin memory T cells (cTRMs). In DNFB‐induced AD mice, the influx of CD8^+^CD69^+^CD103^+^ cTRMs into the epidermis was approximately 60‐fold higher than that of controls, and these CD8^+^ cTRMs are essential for flare neutrophil recruitment during allergen re‐exposure, which is one of the key features of active AD.^140^, ^141^ Besides, a recent study conducted by Braun's research team^142^ revealed that exposure to bacteria elicited a robust adaptive immune response against *S. aureus*, leading to the accumulation of site‐specific γδ and CD4^+^ TRM cells in pre‐existing dermatitis areas. Furthermore, it was observed that these skin‐resident memory T cells specific to *S. aureus* prevented bacterial colonization but exacerbated episodes resembling AD in mice.^142^

#### 2.4.4 Mast cells

Most newly identified drivers of type 2 inflammation, along with their therapeutic targets, involve MCs, eosinophils, T cells, B cells, epithelial cells, and sensory nerves.^143^ MCs, located mainly in the dermis of the skin, contain histamine and are considered to be typical allergic cells.^144^, ^145^, ^146^ MCs are key regulators of IgE‐mediated allergic inflammation, and FcεRI/IgE activation promotes the development of AD.^147^, ^148^ AD skin shows an increased number of MCs, which are present in or near the epidermis and interact with SP containing nerve bundles that show signs of degranulation.^149^, ^150^ Keith and his team^151^ reported that MC infiltration in the skin of AD patients may be regulated by bone marrow‐derived integrin β7^+^MC progenitors. Serhan et al.^152^ reported that house dust mite (HDM) sensitization in mice induces type 2 skin inflammation by activating the MRGPRB2 receptor on MCs, leading to the inhibition of MCs degranulation, which may be an early process in type 2 skin inflammation. Additionally, Chaki's study^153^ revealed that Orai channels contribute to MRGPRX2/B2‐mediated MCs activation. In cockroach allergen‐induced AD, skin inflammation is reduced in MC knockout mice, and MC activation may be regulated by the DC immunoreceptor/ROS/calmodulin kinase II axis.^154^ Interactions between ILC2s and MCs may also contribute to skin inflammation in AD. Toyoshima's team^147^ reported that MC‐derived miR103a‐3p activates ILC2s and promotes a type 2 inflammatory response. Following FcɛRI‐mediated activation, MCs produce PGD2, which induces ILC2s to migrate toward the skin and drives type 2 cytokine production via PGD signaling.^155^, ^156^, ^157^, ^158^ The core signature of AD is marked by the abnormal expression of genes associated with the differentiation of KCs and the transmission of itch signals.^159^ The IgE–MC–histamine axis is the common mechanism underlying itch, and although MC activation and increased histamine release are associated with AD, H1 antihistamines have shown limited efficacy in treating AD‐related itch, indicating the presence of a nonhistaminergic pathway for itch sensation.^160^, ^161^, ^162^ Moreover, The latest research reveals that MCs initiate type 2 inflammation in AD through the activation of MRGPRX2/MRGPRB2, leading to the release of tryptase.^163^ Previous studies have demonstrated an increase in the number of tryptic MCs within AD lesion areas, while chymotrypsin activity shows a decrease. Additionally, it has been found that TSLP production by mouse KCs is regulated by tryptic enzymes. Partial inactivation of chymotrypsin may potentially prolong the survival of proinflammatory cytokines and neuropeptides (e.g., SP), thereby exacerbating inflammation and itching and influencing the development of AD.^164^, ^165^, ^166^, ^167^

#### 2.4.5 Innate lymphoid cells

Innate lymphoid cells (ILCs) are a subset of innate immune cells that resemble T lymphocytes in function and can be classified as ILC1s, ILC2s, or ILC3s based on their surface markers and secreted cytokines, functionally mirroring Th1, Th2, and Th17 cells, respectively.^168^, ^169^, ^170^ ILCs are enriched at the interfaces between the body and the environment and act rapidly at the early stage of the immune response by rapidly responding to signals or inducing cytokines expressed by tissue‐resident cells; in contrast, it takes a few days for the T‐cell response to occur.^168^, ^171^, ^172^ Single‐cell sequencing analysis has revealed a significant increase in ILCs in the skin lesions of AD patients compared with normal skin.^173^ In both the epidermis and dermis layers, ILCs in AD patients can be subdivided into four distinct groups: ILC1/3, ILC2, ILC1/NK, and NK. Notably, the ILC2s subgroup characterized by specific gene expressions such as IL7R, PTGDR2, and GATA3 showed a particularly unique molecular profile.^174^, ^175^ ILC2s are a potential innate source of type 2 cytokines in the pathogenesis of allergic diseases, and ILC2s can be activated by the epithelial‐derived cytokines IL‐33, IL‐25, and TSLP, which in turn stimulate ILC2 production of type 2 cytokines.^176^, ^177^, ^178^, ^179^ Skin‐derived ILC2s express the IL‐33 receptor ST2, which is upregulated during activation, and are abundant in lesional skin biopsies of AD patients.^180^, ^181^ Furthermore, AD skin lesions reveal a rise with high level of IL‐25, TSLP, and IL‐33 receptors.^182^ These findings suggest that ILC2s are most likely involved in the development of AD.^183^ A prior study demonstrated that the overexpression of IL‐33 in KCs induces AD‐like inflammation in mice. This response is predominantly independent of adaptive immune cells and is orchestrated by the involvement of basophils and ILC2s.^184^ Lee's research team^185^ reported that the activation of ILC2s in AD is modulated by TSLP. Inhibition of TSLP production in HDM‐induced AD leads to a decrease in the number of associated ILC2s and mitigates AD‐related symptoms. Despite the notion that AD is a type 2 inflammatory condition, available studies have shown that increased numbers of ILC3s in the skin of AD mice delay the development of AD by neutralizing IL‐17A.^186^, ^187^ However, the overtransfer of ILC3s accelerated AD symptoms, and IL‐17A‐producing ILC3s contributed to the onset and progression of AD by inducing an IL‐33‐driven type 2 immune response. ^186^

#### 2.4.6 Granulocytes

Granulocytes are immune cells with specialized granules in the cytoplasm that consist of neutrophils, eosinophils, and basophils and play important roles in inflammation.^188^, ^189^, ^190^, ^191^, ^192^ Neutrophils are the most abundant granulocytes in the blood and respond rapidly to viral, bacterial and fungal infections.^193^, ^194^ Skin‐infiltrating neutrophils were found to be key triggers of itch in AD, and neutrophil‐induced CXCL10‐dependent CXCR3 receptors promoted itch through the activation of sensory neurons.^195^ In a study encompassing 80 AD patients and 45 healthy controls, AD patients exhibited elevated neutrophil‐to‐lymphocyte ratio (NLR), platelet‐to‐lymphocyte ratio (PLR), and eosinophil counts.^196^ Notably, a positive correlation was identified between NLR (*p* < 0.001) and PLR (*p* < 0.001) with the Scoring Atopic Dermatitis index.^196^ In addition, the relative eosinophil count may correlate with the degree of pruritus, while the NLR may correlate with the degree of inflammation and the size of the affected AD‐lesion.^197^ AD is characterized by Th2 immunity, one of which includes diminished innate immunity and increased dermal infiltration of eosinophils.^198^ Eosinophil influx into the dermis was observed to begin within 2–6 h after using the patch test in patients with AD.^199^ In AD skin, the secretion of IL‐4, IL‐13, and TNF‐α induces the expression of vascular cell adhesion molecule and intercellular adhesion molecule on endothelial cells, facilitating eosinophil recruitment.^200^, ^201^ Specifically, IL‐5 accelerates eosinophil recruitment and activation, while IL‐4 and IL‐13 produced by eosinophils influence T and B cell function, further shaping AD pathology.^202^, ^203^, ^204^ Beyer's team^205^ discovered that eosinophil activation is also regulated by histamine along the IL‐18/IL‐18R axis. The present study revealed that 18 microRNAs in eosinophils were significantly altered in AD patients, suggesting that epigenetic mechanisms could be used to further reveal the underlying mechanisms involved in AD.^206^ Type 2 inflammation, a hallmark of allergic diseases like AD, is distinguished by the accumulation of basophils and ILC2s in inflamed skin lesions, and colocalization of ILC2s and basophils is evident in the skin lesions of AD patients.^207^ Continued depletion of basophils or restriction of basophil‐derived IL‐4 production mitigates skin inflammation and ILC2 accumulation in a murine model of AD. Furthermore, the concerted action of ILC2s and basophils, potentially orchestrated by IL‐33, plays a pivotal role in influencing the development of AD.^184^, ^207^, ^208^, ^209^ In addition, in AD, basophils are spontaneously activated under homeostatic conditions and highly express FcεRI.^210^ Basophil development and peripheral basophil infiltration in AD were dependent on TSLP–TSLP receptor (TSLPR) signaling.^211^, ^212^ A recent study by Wang's team^213^ revealed that basophils are required for acute pruritic episodes in AD‐associated inflammation and that the basophil–leukotriene (LT) axis, which interacts with sensory neurons in the skin, is critical for acute pruritic episodes such as AD. In the MC903‐induced AD model, Pellefigues’ team^212^ observed that basophils, along with IL‐4, may worsen AD through effects on KC differentiation, epidermal proliferation, and M2 macrophage function, contributing to skin barrier dysfunction. Conversely, basophils may also aid in AD recovery by inhibiting proinflammatory cell infiltration. Recent research has indicated that the involvement of a network comprising macrophages expressing IL‐31, TSLP, periostin, and basophils is significant in the manifestation of itching in AD.^214^ Moreover, Difamilast can suppress IL‐4 production from basophils activated in vitro through inhibition of ERK phosphorylation in the skin lesion to ameliorate AD.^215^ The aforementioned findings imply that granulocytes play a significant role in the pathogenesis of AD.

#### 2.4.7 Macrophages

Macrophages are the most abundant skin‐resident immune cells during embryonic development and are present in healthy skin in both M1 macrophages (Mac1) and M2 macrophages (Mac2) states, with Mac2 being significantly increased in areas of AD lesions.^174^ A study combining spatial transcriptomics with single‐cell RNA sequencing identified potential cellular crosstalk between M2 macrophages expressing CCL13 and CCL18 and CD45RO^+^ T lymphocytes in the skin lesion areas of AD patients, promoting type 2 inflammation in AD.^216^ In addition, Zhang's team^217^ found that the expression of Mac2‐secreted CTSC, CCL13, and CCL18 from skin biopsies in AD patients was significantly increased and was proportional to the severity of AD. Furthermore, M2 macrophages are thought to be one of the cellular sources of IL‐31, which is associated with pruritus in AD, and M2 secretion of IL‐31 is influenced by factors such as IL‐4, M‐CSF released by basophils.^214^, ^218^ A growing number of studies have shown that M2‐type macrophages play an important role in the pathogenesis of AD and that inhibition of M2 polarization suppresses the development of AD. Polarization of M2 macrophages is predominantly mediated by IL‐4/STAT6 signaling, regulated by glucose transporter,^219^ circular RNA such as hsa_circ_0004287.^220^ In recent years, Zhu's research team^221^ discovered that defective macrophage autophagy leads to the accumulation of CEBPB, inhibition of the Janus kinases (JAK)1–STAT6 pathway, and impaired M2 polarization, thereby alleviating AD. These findings suggest that targeting macrophage autophagy could be a promising strategy for AD intervention.

#### 2.4.8 Monocytes

Monocytes are circulating single nucleated cells that maintain tissue homeostasis and contribute to various immune responses, and like macrophages are a heterogeneous population.^222^ Under inflammatory conditions, monocytes are recruited to the site of inflammation, where they rapidly differentiate into monocyte‐derived cells that exhibit distinct functional and transcriptional characteristics compared to resident macrophages and conventional dendritic cells.^223^ Monocytes remain in a state of readiness, waiting for cues to be recruited into the dermis, and after differentiation into macrophages, they transform into highly mobile phagocytes capable of initiating an immune response and releasing cytokines via pathogen recognition receptor (PRRs) to promote AD development.^224^ Recently, Fassett's research team^218^ discovered that Il4ra^+^ monocytes, which are capable of driving feed‐forward type 2 inflammatory loops, are enriched in the skin of Il31ra‐deficient HDM dermatitis models. These monocytes may play a role in IL‐31‐mediated neuroimmune pathways and could be involved in paradoxical dermatitis flare‐ups in AD patients undergoing anti‐IL31RA therapy. In addition, IDECs, primarily derived from peripheral blood mononuclear cells, have been reported to express high‐affinity IgE receptors (FcεRI), which induce different T‐cell responses and play a significant role in the pathogenesis of AD.^225^ Recent studies have found that the expression of the TLR2 on peripheral blood monocytes in AD patients is increased.^96^ Monocyte‐derived IDECs are regulated by the endocannabinoid system, including arachidonoylethanolamide, which can activate glycolysis through FcεRI and TLR2.^226^ This activation upregulates the expression of inflammatory cytokines such as TNF‐α and IL‐6, driving diverse T‐cell immune responses and promoting the progression of AD.^227^ Furthermore, a recent study by Seon‐Pil Jin's research team, using single‐cell sequencing analysis of peripheral blood monocytes from 12 patients with severe AD (Eczema Area and Severity Index > 21), found that monocytes with an interferon signature and upregulation of the TLR pathway predominate in severe AD cases.^228^ These monocytes exhibit activation of the innate immune pathway. Besides that, the latest study found that Ly6C^hi^ monocytes are the first major inflammatory cells to infiltrate AD lesions and negatively regulate type 2 inflammation by producing type I IFN.^229^ It is imperative to further elucidate the specific mechanisms underlying monocyte action in AD, taking into consideration the unique characteristics of monocytes.

#### 2.4.9 Other cells

Fibroblasts are the major cell type in the dermis, and their proliferation is regulated by IL‐22.^230^ The previously perceived immunoneutral or quiescent nature of fibroblasts has been revised, as they are now recognized as crucial immune sentinel cells that initiate and regulate immune responses.^231^, ^232^ In addition, inhibiting fibroblast–immunocyte interactions has also been found to facilitate skin regeneration.^233^ The recent application of single‐cell sequencing to the skin of AD patients has revealed a concomitant presence of unique inflammatory fibroblasts in AD lesions.^118^ This suggests that inflammatory fibroblasts may engage in interactions with other immune cells to modulate type 2 inflammation. Research indicates that fibroblasts contribute to skin inflammation by upregulating C‐C motif chemokine ligand 11 (CCL11).^234^, ^235^ Ko's study^236^ demonstrates increased CCL11 expression in fibroblasts from both AD patients and mouse models of AD. Moreover, inhibition of the Ikkb–NF‐κB axis in Prx1 fibroblasts induces CCL11 upregulation, disrupting skin homeostasis and potentially contributing to AD pathogenesis. Furthermore, Prx1 fibroblast dysregulation is a previously unrecognized etiology and suggests that targeting CCL11 to fibroblasts may be a treatment for AD.^236^ In the DNCB‐induced AD model, skin MC‐derived exosomal expression of CXC chemokine ligand 13 (CXCL13) induced histone deacetylase 6 (HDAC6) expression in skin KCs, MCs, and dermal fibroblasts. This heightened the invasive potential of KCs and dermal fibroblasts, leading to increased expression levels of SIRT1 and CXCL13, thereby contributing to the pathogenesis of AD.^237^ Additionally, recent studies have found that periosteal proteins may be associated with a variety of allergic diseases, including AD.^238^, ^239^ IL‐4 and IL‐13 stimulate fibroblasts to express periosteal proteins, further promoting the development of AD.^240^ *S. aureus*‐associated nucleotide‐binding oligomerization domain‐containing protein 2 and TLR2 ligands have been implicated in exacerbating AD‐like symptoms. The interaction of these ligands with dermal fibroblasts promotes the activation of basophils and eosinophils, thereby modulating AD.^241^, ^242^ Additionally, the regulatory influence of IL‐31 and IL‐33 on eosinophils and fibroblasts is noteworthy in this context.^243^, ^244^, ^245^ Suppression of the TNF superfamily molecule TWEAK (TNFSF12) has been shown to diminish the expression of Th2 cells, CCL17, and TSLP in AD‐afflicted skin. In their investigation, Sidler's team^246^ observed an upregulation of the TWEAK receptor Fn14 in KCs and dermal fibroblasts, indicating a potential association between fibroblasts and the regulation of AD through TWEAK. In the mouse model of AD, HMGB1‐induced fibroblast activation plays a crucial role in the development of inflammation and itching. Blocking HMGB1 effectively inhibits fibroblast activation, reducing both inflammation and itching in AD‐like symptoms induced by MC903.^247^ To sum up, fibroblasts play an important role in regulating the development of AD, and targeting fibroblasts and fibroblast‐derived factors offers a promising therapeutic approach for AD treatment.^248^

Furthermore, recent research has revealed that lymphatic vascular endothelial cells (LECs), situated in the skin, serve not only as structural components of the lymphatic system but also perform diverse functions.^249^, ^250^ LECs facilitate the migration of immune cells into lymphatic vessels and drainage to lymph nodes by expressing chemokines and adhesion molecules.^251^ This implies a potential impact of LECs on AD through the modulation of T helper cell differentiation. However, the precise mechanisms involved in this process warrant further investigation.

### 2.5 Cytokines

**FIGURE 5:**
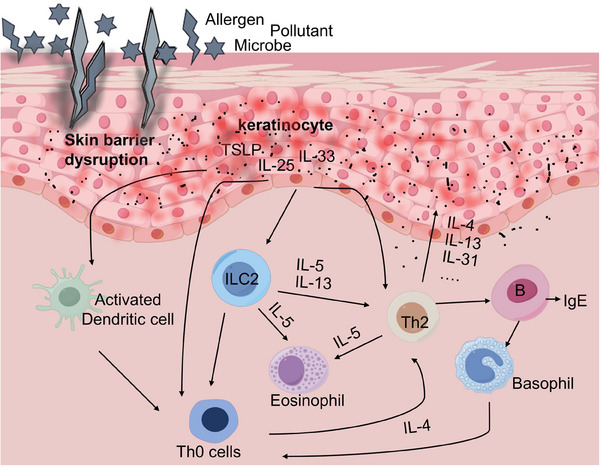
Role of cytokines in AD. Significant quantities of cytokines such as IL‐25, IL‐33, and TSLP to coordinate the infiltration of immune cells into the AD‐lesional skin. The upregulation of these cytokines activates ILC2s, leading to the production of type 2 inflammation‐associated cytokines, including IL‐4. Consequently, this cytokine release influences the infiltration of other cells, such as eosinophils and basophils. This figure was drawn by Figdraw.

Cytokines play a crucial role in the immune system by transmitting signals between cells and inducing various biological responses.^252^ The regulation of AD involves the secretion of various cytokines by KCs, immune cells, and other cells. Notably, type 2 cytokines and epithelial alerting hormones such as IL‐4 and TSLP play a crucial role in the development of AD (Figure 5). This subsection summarizes recent insights into the roles of IL‐4, TSLP, and other cytokines in the pathogenesis of AD.

#### 2.5.1 TSLP in AD

TSLP, a member of the four‐helix bundle cytokine family, has been shown to be a key factor in the maintenance of immune homeostasis and the modulation of inflammatory responses at the mucosal barrier.^253^ In a meta‐analysis, involving 14 studies with 1032 AD patients and 416 controls, reveals elevated serum TSLP levels. Adults show higher TSLP than pediatric AD patients, and TSLP correlates with AD severity, conclusively indicating elevated circulating TSLP in AD.^254^ The present study showed that KCs release TSLP through the ORAI1/NFAT calcium signaling pathway to initiate Th2 and Th22 immune responses.^255^ In addition, Notch deletion in KCs also induces TSLP production,^256^ moreover, signaling between epithelial cells and innate immune cells occurs through signals emitted by TSLP.^257^ Furthermore, Siracusa et al.^258^ findings support that TSLP‐induced progenitor cells differentiate into effector cells, which include macrophages, dendritic cells, and granulocytes and promote type 2 cytokine‐mediated inflammation, similar to AD. A recent study found that obesity triggers activation of the CD36–SREBP1–TSLP axis in KCs, leading to epidermal lipid disorders and increased AD‐like inflammation.^259^ MC903 was applied to the ears of TSLP‐overexpressing or TSLP‐knockout mice to induce AD, and TSLP overexpression was shown to affect not only Th2 differentiation but also TFH cell differentiation and IL‐4 production.^91^ The recognition of HDMs as a crucial sensitization route in AD patients emphasizes the distinctive requirement of TSLP in epicutaneous or dermacutaneous HDM‐induced Th2/Tfh responses.^260^ Pruritus is one of the main features of AD, and Wilson et al.’s^257^ study revealed that TSLP acts directly on a subset of TRPA1^+^ sensory neurons to trigger intense itchy behavior, suggesting that KC‐derived TSLP could serve as a therapeutic target for AD‐related pruritus. In addition, TSLP–TSLPR signaling plays a key role in AD‐like inflammation and TSLP mediates its effects through a heterodimeric receptor complex comprising IL‐7Rα (encoded by IL7R) and TSLPR (encoded by CRLF2).^261^, ^262^, ^263^ Berna's team^264^ used next‐generation sequencing to analyze TSLP and IL7R genes, finding that the TSLP variants rs10073816, rs61423440, and rs60340825 were linked to more persistent forms of AD, with IL7R variants potentially modulating the role of TSLP variants. Tezepelumab (AMG‐157/MEDI9929) is a first‐in‐class human IgG2λ monoclonal antibody that inhibits the action of TSLP, which has been tested in a recent clinical trial, and the results revealed that AD patients have improved symptoms over placebo.^265^, ^266^

#### 2.5.2 IL‐33 in AD

IL‐33 is an inflammatory cytokine of the IL‐1 family, abundantly expressed both during homeostasis and inflammation and usually released upon tissue injury, considering as an alarmin.^267^, ^268^ It was reported that IL‐33 secretion by KCs is dependent on extracellular ATP/P2Y2 signaling, thereby stimulating transactivation of the epidermal growth factor receptor, which in turn induces IL‐33 synthesis and secretion.^269^ IL‐33, as well as the receptor ST2 and the coreceptor IL‐1RAcP, are highly expressed in the skin of AD patients, suggesting that IL‐33 may be closely associated with the development of AD.^270^ Al Kindi's^271^ laboratory infected mice with *S. aureus* and demonstrated that *S. aureus* induced rapid release of IL‐33 from human KCs, which in turn drove the development of AD. Peng and colleagues^272^ developed an AD model by subcutaneously injecting mice with 2,4‐dinitrochlorobenzene (DNCB) for 33 days, combined with anti‐mouse IL‐33 antibody treatment. They observed reduced serum IgE levels and symptom improvement, suggesting IL‐33's involvement in AD.^272^ Moreover, the induction of dermatitis resembling AD was observed in a transgenic mouse model characterized by the overexpression of IL‐33 in the skin.^273^ Furthermore, IL‐33 activates various immune cell types associated with type‐2 immunity and allergic inflammation, including ILC2s, Th2 cells, and basophils.^274^, ^275^ Imai's team^276^ reported that after constructing IL‐33‐overexpressing transgenic mice, basophils in IL‐33‐overexpressing transgenic mice accumulated in the AD lesion areas of the mice and that when basophils and type 2 innate lymphocytes (ILC2s) were depleted, the development of AD‐like inflammation was almost completely suppressed in IL‐33 transgenic mice. These findings suggested that IL‐33 may regulate the development of AD by modulating basophils and ILCs. In addition, a recent study revealed that IL‐33 regulates AD by modulating the expression of sebum and its microbial metabolite propionate on the surface of AD skin in MC903‐induced AD‐like mice; however, knockdown of IL‐33 or the IL‐33 receptor ST2 did not prevent the development of AD‐like skin inflammation.^277^, ^278^ Although further research is required, these studies suggest that IL‐33 secretion by KCs promotes basophil and ILC2 accumulation in AD lesion areas, thereby contributing to AD development. Additionally, dysregulation of the sebum–microbial metabolite–IL‐33 axis may play a significant role in AD and other inflammatory skin diseases.

#### 2.5.3 IL‐25 in AD

IL‐25, also known as IL‐17E, is a cytokine of the IL‐17 family and is produced by several types of immune cells, such as T cells, dendritic cells, and group 2 innate lymphocytes.^279^ IL‐25 has been recognized to act as epithelial barrier damage alarmin.^280^ A large amount of IL‐25 is derived from epithelial cells, and IL‐25 is a barrier‐surface cytokine whose expression is dependent on external environmental factors and may lead to inflammatory diseases when upregulated.^279^ Actually, IL‐25 was found to strongly increase production of cytokines associated with type 2 immunity.^281^ In mouse skin, IL‐25 activates the Act1–JAK1/2–STAT3 pathway, which leads to KC proliferation and the generation of inflammatory cytokines and chemokines.^282^ In addition, IL‐25 generates Th2‐associated cytokines IL‐4, IL‐5, and IL‐13,^283^ as well as Th2‐like characteristics, and the concentration of IL‐25 in serum is considerably higher in individuals with moderate to severe AD.^284^ Leyva‐Castillo's team^182^ used OVA to construct acute and chronic AD models in mice with KC‐specific knockout of IL‐25R or IL‐13‐driven transfection of green fluorescent protein. They discovered that KC‐derived IL‐25 is required for acute allergic skin inflammation and that IL‐25 acts directly on ILC2s to drive acute allergic skin inflammation and IL‐13 production, which activates epidermal hyperplasia and CD4^+^T cell accumulation in acute and chronic antigen‐driven allergic skin inflammation.^182^ In addition, MK Aktar and his team reported that IL‐25 upregulates the mRNA and protein expression of the pruritogenic factor endothelin‐1 in HaCaT cells in a concentration‐ and time‐dependent manner, which may be one of the mechanisms leading to itch in AD.^281^ Previous studies have reported that the addition of IL‐25 reduces filipin synthesis in the skin,^285^ suggesting that IL‐25 may impair the skin barrier not only by stimulating cytokines expression, such as Th2 cells, but also by downregulating the synthesis of filipin in the skin, which provides a rationale for the link between inflammatory responses and impaired skin barrier function. IL‐25 is a cytokine with both proinflammatory and anti‐inflammatory effects, and according to new clinical data, the expression of IL‐25 is lower in the cord blood of infants with AD than in that of infants without AD.^286^ These findings suggest that IL‐25 may regulate AD progression by affecting the skin barrier or ILC2s, but the role of IL‐25 in AD and the specific signaling mechanisms involved need to be further explored.

#### 2.5.4 IL‐4 in AD

IL‐4 has been identified as a pivotal cytokine in triggering the Th2 response, contributing to the recruitment and production of IgE, along with other immune markers and cells, which plays a crucial role in the development of atopic diseases.^287^, ^288^ The direct impact of the cytokine IL‐4 on the epidermis in patients with AD has been suggested. These effects encompass the potential inhibition of terminal differentiation, which may contribute to excessive proliferation of KCs, as well as the induction of spongiosis.^289^, ^290^, ^291^ Moreover, IL‐4 hinders lipid synthesis, suppresses the production of antimicrobial peptides, and facilitates binding and colonization by *S. aureus*.^288^ According to a recent study conducted by Takahashi's team,^292^ it has been discovered that the topical application of a phosphodiesterase 4 (PDE4) inhibitor effectively enhances AD treatment by suppressing basophil IL‐4 production. Additionally, IL‐4 has been shown to augment neurogenic itch by directly impacting pruritogenic sensory neurons and interacting with IL‐31, moreover the presence of IL‐4 in AD skin leads to a decrease in the synthesis of antimicrobial peptides, which consequently increases the vulnerability of the skin to invasion by foreign pathogens.^291^

## 3 EXPERIMENTAL MODELS IN AD

Understanding of the physiopathology of AD has been greatly advanced through the use of animal models and in vitro experimental systems. The pathological mechanisms, drug efficacy, and skin barrier function of AD are extensively investigated using in vitro and in vivo experimental models. Given that each AD mouse model possesses distinct strengths and limitations, comprehending the distinctive attributes of specific models will significantly contribute to the study's overall success. Therefore, in this subsection, our focus lies on summarizing the commonly employed experimental models for studying AD.

### 3.1 In vivo experimental model of AD

**FIGURE 6:**
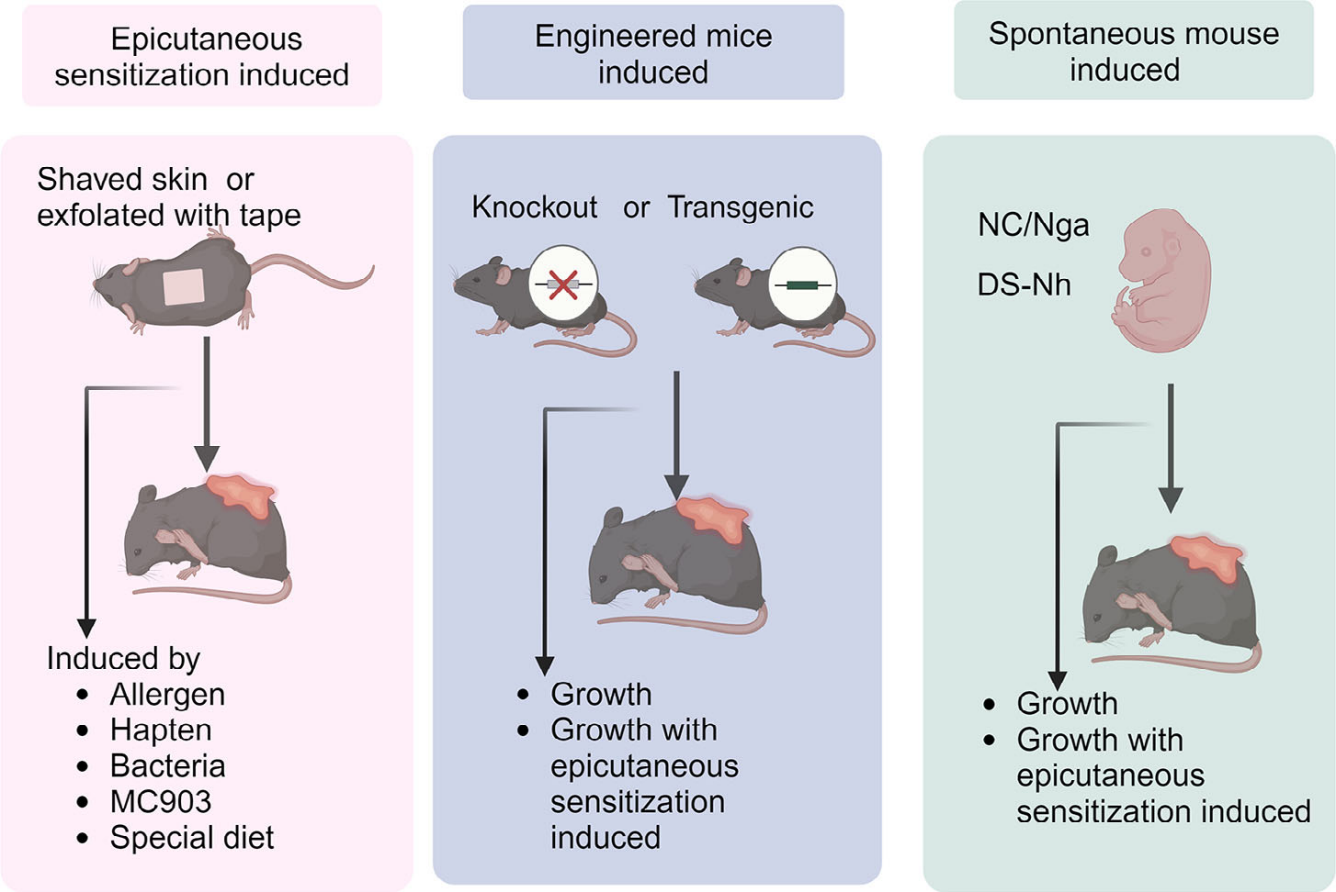
A summary of in vivo models for AD. Numerous mouse models have been created for the study of AD. In addition to generating humanized models of AD through the transplantation of human immune cells or skin tissue, different categories of AD models exist. These include models induced by topically applying sensitizers on the skin, transgenic mice that exhibit either excessive or deficient levels of specific molecules relevant to AD, and mice that spontaneously develop cutaneous lesions resembling those seen in individuals with AD. This figure was drawn by BioRender.com.

The majority of in vivo experimental models employ mice to replicate the symptoms, immune responses, and pathological characteristics associated with AD. Given their importance in preclinical research on new therapeutics and for understanding AD pathobiology, a wide variety of mouse models have been developed. Additionally, humanized AD models have been constructed by transplanting human immune cells or skin tissue.^293^, ^294^ AD mice models can be classified into three categories: those induced by the topical application of sensitizers to the skin, transgenic mice that either overexpress or lack specific molecules relevant to AD, and mice that spontaneously develop AD‐like cutaneous lesions.^27^ The in vivo experimental models of AD are summarized in Figure 6.

#### 3.1.1 AD models induced by epidermal sensitization

It has been reported that an AD model, induced by shaving the back skin of mice and repeatedly exfoliating with 3M tape to simulate the scratching‐induced skin damage seen in AD patients, followed by repeated epidermal sensitization with OVA, can be used in five mouse strains, including BALB/c and C57BL/6.^295^ Furthermore, the use of HDM allergen in the epidermal contact (EC)‐induced AD model is supported by clinical studies, demonstrating a significant correlation between this approach and human AD.^296^ In recent years, Leyva‐Castillo's^297^ laboratory has developed a mouse model of AD‐like skin inflammation induced by repeated epicutaneous sensitization (EC) of tape stripped skin with OVA or peanut antigens, which is similarly with human AD, and widely used in investigating allergic skin inflammation in AD. In addition, AD models can be induced by hapten such as DNFB, oxazolone, trinitrochlorobenzene (TNCB), and DNCB.^298^ These hapten sensitization models are widely employed to evaluate the pathogenic mechanisms and potential therapies for AD due to their reproducibility, predictability, cost effectiveness, efficiency, and their resemblance to the pathogenesis observed in human AD patients.^27^ Moreover, calcipotriol (MC903), a synthetic vitamin D3 analogue, upregulates TSLP when applied topically to mice, resulting in AD‐like lesions, while, this model does not induce TSLP expression in healthy human skin, nonlesional AD skin, or nonhuman primate skin, suggesting that it may be unsuitable for studying AD pathogenesis in humans.^299^, ^300^ Nevertheless, due to its reproducibility and ease of use, it is still widely employed to evaluate potential AD drug candidates.^301^, ^302^ Additionally, AD models can be constructed by repeatedly sensitizing tape‐exfoliated skin with *S. aureus* or through repeated intragastric sensitization with allergens such as milk or peanuts.^303^, ^304^

#### 3.1.2 AD models induced by genetically engineered mice

In addition to repeated allergen sensitization, AD can also be modelled through genetic engineering techniques, by creating mice with specific genes either knocked out or overexpressed. An increasing number of studies have shown that the overexpression of type 2 cytokines, such as IL‐4, IL‐13, IL‐33, IL‐18, and TSLP, by KCs activates ILC2s and promotes the development of AD‐like skin lesions.^305^ Lawrence S. Chan's team discovered that mice overexpressing IL‐4 in basal KCs developed spontaneous itching and chronic dermatitis by 4 months of age, progressively exhibiting features characteristic of human AD.^306^ Additionally, transgenic mice overexpressing IL‐31 were found to develop severe itching, alopecia, and skin lesions, showing signs of dermatitis similar to human AD as early as 2 months of age.^307^ Moreover, a disintegrin and metalloproteinase 17 (ADAM17), a transmembrane protease responsible for cleaving cell membrane‐anchored proteins, also plays a crucial role. ADAM17 deficiency leads to the development of eczematous dermatitis accompanied by naturally occurring dysbiosis, resembling that observed in AD.^308^ Similarly, Apolipoprotein C1 transgenic mice not only exhibit disrupted serum lipid levels but also spontaneously develop severe dermatitis resembling AD as they age.^309^, ^310^ While recent advances in genetic engineering have accelerated our understanding of the biological significance of targeted genes in vivo, the generation of genetically engineered mice is both time‐consuming and expensive and is often applied to explore the role and mechanisms of cytokines in AD.

#### 3.1.3 Spontaneous models of AD

The Nc/Nga mice are the first reported autogamous mouse strain capable of spontaneously developing AD‐like symptoms under conventional breeding conditions.^311^ These mice carry a mutation on chromosome 9 and exhibit pronounced type 2 immune responses.^312^ Currently, haptens such as DNCB, TNCB are used as sensitizers in conjunction with NC/Nga mice to induce AD under specific pathogen‐free conditions and are widely employed for the preliminary screening of potential anti‐AD drugs.^313^, ^314^ Moreover, *Flg^ft/ft^* mice exhibit spontaneous dermatitis with skin lesions similar to human AD, including desquamation, erythema, pruritus, and vesicles followed by edema, making them a promising model for preclinical studies focused on managing AD in children.^315^ Additionally, DS‐Nh mice can spontaneously develop dermatitis characterized by the infiltration of Th2‐type inflammatory cells and cytokines in skin lesions when housed in a regular environment. This phenotype resembles *S. aureus*‐induced AD in humans, offering a valuable model for investigating the role of *S. aureus* in AD pathogenesis.^316^

### 3.2 In vitro experimental model of AD

**FIGURE 7:**
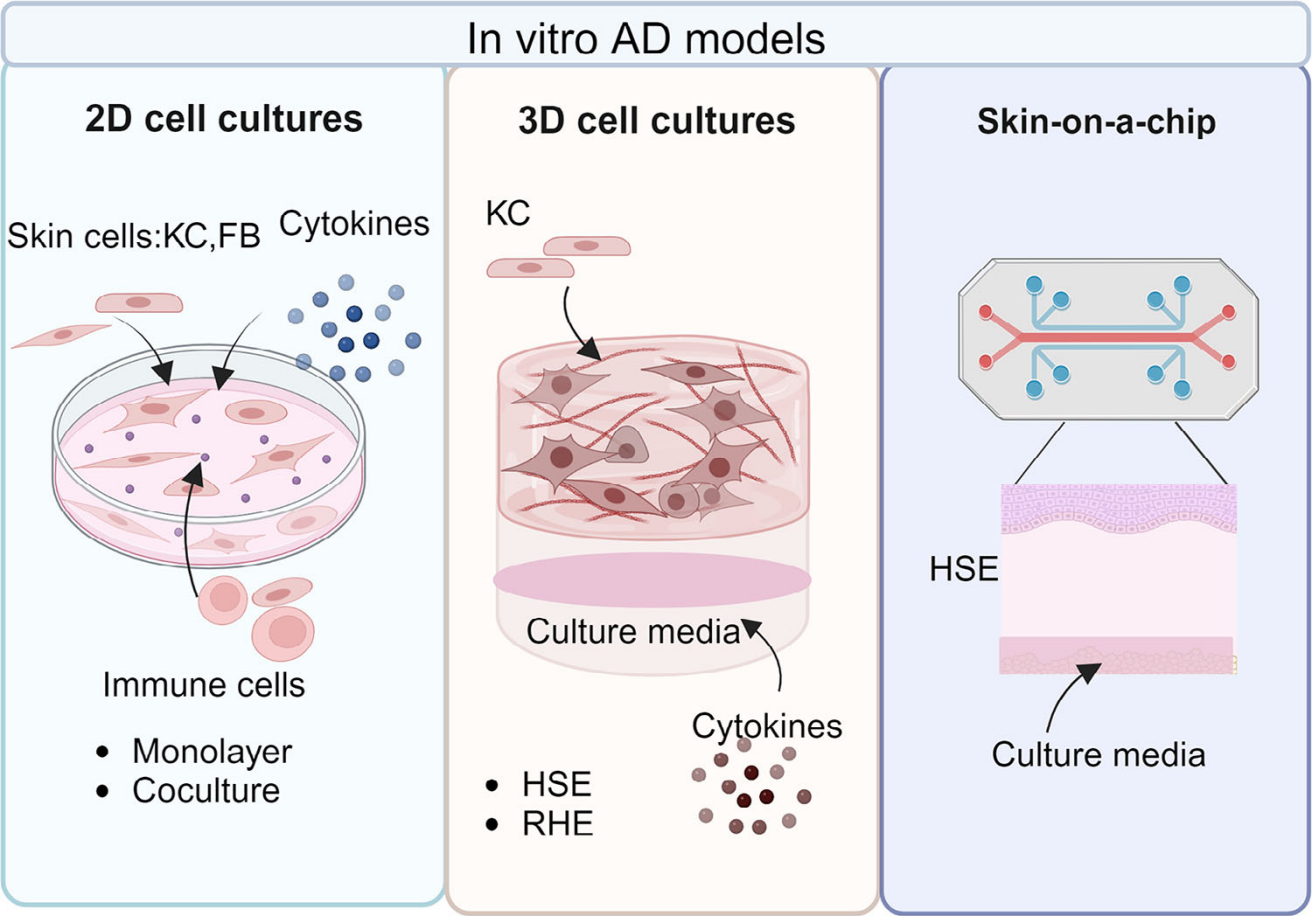
A summary of in vitro models for AD. To gain a comprehensive understanding of the inflammatory process underlying AD and conduct thorough investigations, various in vitro models have been developed. These include 2D monolayer and coculture systems, advanced 3D skin models including reconstructed human epidermis (RHE) and full‐thickness human skin equivalents (HSE), as well as innovative skin‐on‐a‐chip systems that accurately represent skin. This figure was drawn by BioRender.com.

In vitro experimental models primarily utilize cell culture techniques to investigate the molecular mechanisms of AD and for drug screening. The simplest in vitro models of AD consist of either one type of cell in a monolayer or a coculture of multiple cell types, such as KCs or immune cells derived from AD patients or AD models, which allow for the analysis of processes involved in the etiology of epidermal inflammatory diseases and accurate assessment of specific cell responses to various challenges.^317^, ^318^ However, these models fail to produce effective barriers or account for the complex structures and interactions within tissues. To generate an effective epidermal barrier, three‐dimensional skin models have been developed by exposing KCs to the air‐liquid interface. Two common types of 3D skin models used in AD research are reconstructed human epidermis (RHE) and full‐thickness human skin equivalents (HSE).^319^ RHE consists exclusively of KCs cultured on inert polycarbonate filters at the air‐liquid interface, while HSE is built upon a dermal‐like matrix, which is first organized and then seeded with KCs that undergo epidermal stratification and keratinization upon air exposure.^320^ In vitro induction of AD in both RHE and HSE requires the addition of specific amounts of cytokines such as IL‐4 and IL‐13.^320^ These 3D skin‐based models closely mimic the in vivo characteristics of human epidermis, making them widely used for studying skin barrier integrity and the effects of external stimuli. Additionally, the relatively new Skin‐on‐a‐Chip model offers all the advantages of the aforementioned 3D models while also having the potential to replicate the vascular and molecular environment of the body, providing an excellent in vitro model for probing the mechanisms of AD as well as for high‐throughput drug screening (Figure 7).^318^

## 4 THERAPEUTIC INTERVENTIONS

The management approaches for AD ought to be customized according to the unique attributes and advancement of the illness in each patient, with a primary focus on symptom relief, inflammation control, and the maintenance of remission.^321^ Recent advances in understanding AD pathogenesis have led to the development of emerging therapeutic options, offering new hope for disease management. While novel agents, such as biologics, have significantly advanced AD treatment, traditional therapies remain the cornerstone of clinical care. In this section, we will review both traditional and novel treatments for AD, discussing their mechanisms of action, efficacy, and limitations.

### 4.1 Traditional treatment

#### 4.1.1 Topical therapies

The selection of treatment for AD is contingent upon various factors, including the anatomical location of the disease and severity of the disease. In most instances characterized by mild symptoms, topical therapy involving corticosteroids and calcineurin inhibitors generally proves to be adequate. Topical corticosteroids (TCS) are widely recognized as first‐line anti‐inflammatory agents and can be categorized into different potency levels based on vasoconstriction tests.^322^ Drug selection should take into account disease severity, patient age, and lesion location. Appropriate and intermittent use of TCS is essential to avoid local adverse reactions, such as skin thinning, subcutaneous bleeding, capillary dilation, and abnormal pigmentation.^32^ Although systemic adverse effects of TCS are extremely rare, they can include hypothalamic–pituitary–adrenal axis suppression.^323^

Topical calcineurin inhibitors (TCIs) represent a distinct class of topical anti‐inflammatory medications, with tacrolimus and pimecrolimus being the most frequently employed agents. The therapeutic efficacy of TCIs is achieved through the inhibition of calcineurin‐dependent T cell activation and proliferation.^324^ Unlike TCS, TCIs do not cause cutaneous atrophy, rendering them safe for application on delicate areas such as the face, neck, and other regions with thin skin; however, they may elicit a sensation of burning or itching.^325^ The current approach involves the integration of local treatment with other therapeutic modalities to enhance its efficacy.

#### 4.1.2 Systemic traditional therapies

Compared with topical therapies, systemic administration of oral medications offers a more convenient therapeutic option. Furthermore, the utilization of local treatment may not always be feasible for patients with chronic and severe AD, necessitating the implementation of systemic interventions such as antihistamines, oral corticosteroids, and immunosuppressants like cyclosporine and methotrexate.

Antihistamines are primarily utilized for alleviating pruritus symptoms, particularly during nocturnal periods, and patients experiencing severe sleep disturbances due to itching may benefit from short‐term, intermittent use of oral sedative antihistamines.^326^ However, topical antihistamines carry a risk of contact dermatitis, and there are currently no specific guidelines regarding their use in the treatment of AD.

Although systemic corticosteroids have been approved by the United State Food and Drug Administration (US FDA) for the treatment of AD, their use should be restricted to the short‐term management of acute episodes.^327^, ^328^ Systemic steroids are associated with a wide range of adverse effects, including hypertension, glucose intolerance, weight gain, decreased bone mineral density, avascular necrosis of the femoral head, adrenal suppression, mood instability, and immunosuppression; hence, they are really not recommended for long‐term therapy.^329^

Nonspecific immunosuppressants such as cyclosporine and methotrexate are commonly employed in patients with refractory AD due to their notable therapeutic efficacy.^330^ These agents reduce inflammation by suppressing the immune response, making them the most effective treatment option for AD until the advent of biologics. However, prolonged use of immunosuppressants can lead to significant adverse effects; for example, cyclosporine is associated with nephrotoxicity,^325^ while methotrexate is linked to gastrointestinal and myeloid toxicity, as well as hepatotoxicity.^331^, ^332^ Consequently, long‐term administration of these drugs requires careful monitoring.

### 4.2 Emerging therapies

Personalized treatment options are determined by factors such as efficacy, potential side effects, mechanism of action, and the preferred method of administration. While traditional treatments for AD, such as TCS and immunosuppressants, have demonstrated some efficacy, their associated side effects and potential for long‐term dependency leave significant gaps in meeting patients’ treatment needs. Recently, advancements in understanding the pathogenesis of AD have led to the emergence of a variety of novel therapeutic approaches, offering renewed hope for more effective management of this condition. This subsection aims to provide a concise overview of these innovative treatment modalities, exploring their mechanisms of action, effectiveness, and prospects for clinical application.

#### 4.2.1 Molecular inhibitors and biologic therapies

The main objectives of AD treatments are to enhance the overall well‐being of patients, alleviate the intensity of pathological symptoms, minimize the risk of acquiring new infections, and effectively manage the condition in order to facilitate patients' full engagement in their daily activities.^56^ While, according to the European Task Force on Atopic Dermatitis and the International Eczema Council, major international guidelines are increasingly discouraging the use of systemic corticosteroids, cyclosporin, methotrexate, mycophenolate mofetil, and azathioprine.^333^

The selective targeting of specific signaling pathways and enzymatic activities by small molecular inhibitors presents a promising strategy for the management of AD, thus attracting significant attention within the research community. JAKs play a crucial role in regulating the intracellular signaling of key cytokines associated with AD. Inhibiting JNK can reduce AD symptoms by disrupting the JNK–STAT signaling pathway.^334^ Clinical trials have demonstrated that these drugs offer significant therapeutic effects in alleviating AD symptoms and are relatively safe, making them suitable for the long‐term management of moderate to severe AD patients.^335^ Notably, the JAK inhibitor delgocitinib was recently approved in Japan for the treatment of moderate to severe AD.^336^ An assessment was conducted on tezepelumab, a novel monoclonal antibody that hinders TSLP, in a Phase IIa study involving individuals diagnosed with moderate‐to‐severe AD.^265^ Additionally, PDE4 is involved in the degradation of cyclic adenosine monophosphate (cAMP) in inflammation and immune cells, inhibiting PDE4 can elevate cAMP levels, reducing T cell activation and inflammatory factor production, thereby exerting an anti‐inflammatory effect.^337^ Consequently, PDE4 is an important target for both local and systemic treatment of AD. The main PDE4 inhibitors for treating AD include apremilast and crisaborole, the latter of which is a topical cream that has been approved by the US FDA for the treatment of AD in patients over 3 months old.^337^, ^338^ Moreover, As outlined in our review, a hallmark of AD is profound pruritus, with the LT axis playing a crucial role in acute pruritic episodes by interacting with sensory neurons in the skin IL‐13 acts as a direct enhancer in various pruritic and neuroactive pathways and clinical studies indicate that sustained administration of IL‐13 receptor inhibitors, such as lebrikizumab and tralokinumab, provides relief from itch in patients with AD.^339^, ^340^, ^341^

In addition to the utilization of small molecule inhibitors, biologic therapy has also exhibited remarkable potential in the management of AD in recent years. clinical studies have demonstrated that the IL‐31R inhibitor nemolizumab effectively reduces itching in AD patients.^342^ In addition, dupilumab, an IL4R‐blocking antibody, has shown clinical efficacy in the treatment of AD, and it's noteworthy that besides conjunctivitis/blepharitis, emerging dermatitis in the head and neck area is now recognized as a significant side effect, with an incidence of up to 10%.^343^ The aromatic hydrocarbon receptor (AHR) is a ligand‐activated transcription factor that, upon activation, enhances skin barrier function and mitigates symptoms of AD, thereby prompting the development of AHR agonists such as Tapinarof.^344^, ^345^ Additionally, OX40, a member of the TNF receptor superfamily, is expressed following T cell activation and can stimulate Th2 cells to produce cytokines, thereby promoting the development of AD.^346^ Currently, biological agents targeting the OX40 receptor, such as amlitelimab and rocatinlimab, have demonstrated significant improvements compared with placebo in Phase II clinical trials.^347^, ^348^ Dupilumab, an IL‐4R receptor inhibitor, is the first biologic agent targeting the IL‐4 and IL‐13 signaling pathways, demonstrating favorable efficacy and safety across diverse populations and establishing itself as a critical treatment option for AD.^16^, ^349^ As of 2023, China has approved dupilumab and JAK inhibitors for patients with AD who have a minimum disease duration of 6 months.^34^ Recent studies have demonstrated that IRAK4 inhibitors can alleviate AD‐like skin inflammation induced by MC903 and DNFB.^350^, ^351^ Furthermore, a Phase I clinical trial of the IRAK4 inhibitor KT‐474 found it effective in reducing symptoms in patients with AD.^352^ Additionally, Table 1 summarizes the mechanisms and progress of molecular inhibitors and biologic therapies in AD.

**TABLE 1:** The molecular inhibitors and biologic therapies in AD.

Name	Mechanisms	Target	Status of investigation	Application	Possible Adverse effects	Reference
Abrocitinib	Selectively blocking one or more of the JAK family kinases JAK1, JAK2, JAK3 and TYK2, leading to the suppression of various inflammatory pathways.	JAK1	Phase III, In progress	For individuals aged 12 years and older.	Acne; Nausea; Herpes zoster; Nasopharyngitis; Gastrointestinal disorders; Thrombocytopenia; Pneumonia; Herpetic eczema.	353, 354
Baricitinib		JAK1/JAK2	Phase III, In progress	Approved in Europe for adults and children aged 2 years and older with moderate to severe AD.	Blood creatinine phosphokinase elevation; Herpes zoster; Nasopharyngitis.	353, 355, 356
Upadacitinib		JAK1	Phase III, In progress	For individuals aged 12 years and older.	Acne;Nasopharyngitis;Upper respiratory tract infection; Headache; Creatine phosphokinase elevation; Oral herpes; Folliculitis; Cough; Nausea; Herpes zoster.	162, 353, 355, 357
Delgocitinib		JAK1, JAK2, JAK3, TYK2	Phase II, Completed	Children, adults	Nasopharyngitis; Blood creatinine phosphokinase elevation; Contact dermatitis; Folliculitis; Acne.	162, 358, 359, 360
Ruxolitinib		JAK1, JAK2	Phase III, In progress	Short‐term and intermittent chronic treatment for mild‐to‐moderate AD in non‐immunocompromised patients aged 12 and older.	Nasopharyngitis.	162, 358, 359, 361
Dupilumab	The IL‐4 receptor inhibitor can suppress the production of IL‐4 and IL‐13	IL‐4R	Phase III, In progress	For children aged 6 to 11 years, and for moderate to severe AD in adults, adolescents, and children aged 1 year or older.	Nasopharyngitis; Conjunctivitis; Keratitis; Injection‐site reaction; HSV infection; Upper respiratory tract infections; Headache; Oral herpes.	288, 353, 362
Nemolizumab	Inhibiting the activity of IL‐31, thus preventing the transmission of itch signals to cutaneous neurons.	IL‐31R	Phase III, In progress	For moderate‐to‐severe pruritus	Nasopharyngitis; Blood creatine phosphokinase increased; Contact dermatitis; Influenza; Urticaria; Acne; Cellulitis; Headache; Dental caries; Upper respiratory tract inflammation; Gastroenteritis.	342, 363
Asivatrep	A TRPV1 antagonist can inhibit IL‐31‐induced itching in a serpin E1‐dependent manner.	TRPV1	Phase III, In progress	For patients were aged 12 years or older, with mild‐to‐moderate AD.	Nasopharyngitis; Urticaria; Burning sensation; Rhinorrhea.	280, 364
Tapinarof	Activation of AhR signaling pathways modulates gene expression, leading to the downregulation of Type 2 inflammation, normalization of the skin barrier, and reduction of oxidative stress.	AhR	Phase III, In progress	For adolescents and adults, a Phase III clinical trial is ongoing.	Nasopharyngitis; Upper respiratory tract infection; Worsening of AD; Folliculitis.	280, 359, 365
Tralokinumab	Selectively blocking IL‐13 signalling.	IL‐13	Phase III, In progress	For adults with moderate to severe AD.	Injection site reactions; Upper respiratory tract infections; Conjunctivitis; Headache.	353, 366
Lebrikizumab				For moderate‐to‐severe AD aged 12 year or older.	Conjunctivitis; Keratitis; Nonmelanoma skin cancer; Herpes infections.	353, 367
Crisaborole	A topical PDE inhibitor that prevents the release of inflammatory cytokines by increasing intracellular cAMP.	PDE4	Phase III, In progress	For children aged 3 months and older with mild‐to‐moderate AD.	Application site pain;Skin and subcutaneous tissue disorders;Dermatitis contac;Erythema.	368, 369, 370
Roflumilast	Inhibiting PDE4		Phase IIa, Completed.	While approved for plaque psoriasis, some healthcare providers may consider off‐label use of roflumilast for patients with mild‐to‐moderate AD who experience intolerance to crisaborole due to local side effects.	Hyperkalemia;Migraine;Ncreased alanine aminotransferase level; anemia;Application site pain; Diarrhea; Insomnia.	371
Difamilast			Phase III, Completed.	For individuals aged 2 years or older with mild to moderate AD.	Nasopharyngitis.	358, 372
Rocatinlimab	Blocking OX40 downregulates Th2, Th1/Th17, and Th22 inflammation, inhibiting pathogenic T cells and associated inflammation	OX40	Phase II, Completed	For moderate‐to‐severe AD	Nasopharyngitis.	130, 348, 373
Benralizumab	It interfered the activity of IL‐5 and blocked IL‐5Rα	IL‐5	Phase II, In progress	For moderate to severe AD	COVID‐19 infection; Upper respiratory tract infection; Headache; Swelling of the lymph nodes; Conjunctivitis	374
Tezepelumab	It is a human IgG2 monoclonal antibody that blocks the interaction between TSLP and its receptor complex	TSLP	Phase IIa, Terminated	For moderate to severe AD	Nasopharyngitis; Injection‐site erythema	265
Secukinumab	It is an IL‐17A inhibitor	Il‐17A	Phase II, Terminated	For moderate to severe AD	Worse AD; Orbital cellulitis; Upper respiratory infection; Streptococcal pharyngitis	375
Ligelizumab	It is a humanized IgG1k monoclonal antibody specifically designed to target the constant region of the heavy chain of IgE	IgE	Phase II, Completed	For moderate to severe AD	Headache; Urticaria	376
Fezakinumab	It is a human IgG1‐lambda monoclonal antibody that binds directly to IL‐22, preventing the formation of the IL‐22/IL‐22 receptor complex and inhibiting downstream signaling.	IL‐22	Phase II, Completed	For moderate to severe AD	Upper respiratory infection	377, 378, 379
RPT193	CCR4 small molecule antagonists can inhibit the migration and downstream activation of Th2 cells	CCR4	Phase I, Completed	For moderate to severe AD	Headache; Nausea; Generalized rash of moderate severity	126
Etokimab	It is a humanized anti‐IL‐33 monoclonal antibody, which inhibits IL‐33 and suppresses inflammation and skin barrier in AD	IL‐33	Phase IIb, In progress	For moderate to severe AD	Upper respiratory tract infection; Urinary tract infection	380, 381
Spesolimab	It is a humanized monoclonal IgG1 that specifically binds to IL‐36R, blocking ligand activity and inhibiting pro‐inflammatory signaling downstream.	IL‐36	Phase II, Completed	For moderate to severe AD	Nasopharyngitis; Folliculitis; Upper respiratory tract infection	382
Bermekimab	Specific blockade of IL‐1α activity and effectively inhibits the inflammatory process	IL‐1α	Phase II, Terminated	For moderate to severe AD	Worse AD; Nasopharyngitis; Upper respiratory tract infection	383
KT‐474	Inhibit the signaling of all IL‐1 family cytokines and almost all TLRs	IRAK4	Phase I, Completed	For moderate to severe AD	Mild to moderate headache; Palpitations; Nausea;Vomiting; Diarrhea	352

Abbreviations: AhR, Aryl hydrocarbon receptor; TRPV1, Transient receptor potential vanilloid subfamily V member 1; TYK2, Tyrosine kinase 2.

#### 4.2.2 Microbial therapy

The skin microbiota plays a crucial role in the pathogenesis of AD, particularly concerning the excessive growth of *S. aureus*, which is strongly correlated with the exacerbation of AD symptoms. Although randomized clinical trials have shown that antibiotics effectively inhibit *S. aureus* growth and significantly alleviate AD symptoms, uncertainty remains regarding their overall clinical efficacy in treating this condition.^384^ Based on this understanding, microbiotherapy is emerging as a promising therapeutic approach. Oral probiotics, particularly formulations containing *Lactobacillus* and *Bifidobacterium*, have shown potential in treating allergic diseases, especially AD, as they can help restore the balance of skin microorganisms and alleviate AD symptoms.^385^, ^386^ A 12‐week treatment of children aged 1–12 years with moderate AD using tyndallizate *Lactobacillus rhamnosus* IDCC 3201 (RHT3201) resulted in a significant reduction in AD scores, as well as in eosinophil cationic protein and IL‐31 levels.^387^ However, due to the risk of intestinal luminal transfer to the bloodstream in vulnerable populations, such as infants, the use of heat‐inactivated probiotics is now recommended instead of live probiotics.^385^ Furthermore, topical probiotics have been shown to improve the ecology of the skin's microbial community, reduce the adhesion and survival of certain pathogens, and inhibit the development of AD.^388^ While specific probiotics can decrease the inflammatory response associated with AD and enhance skin barrier function, their mechanisms of action, as well as long‐term effects and safety, require further validation.^389^

### 4.3 Adjuvant therapy

In addition to medication, complementary therapies play a vital role in the comprehensive management of AD. Skin care measures, including emollients and moisturizers, moisturize the skin, help repair the skin barrier, reduce skin inflammation, and regenerate skin structure.^51^, ^56^ Nonpharmacological topical moisturizers serve as the cornerstone of AD management. Effective formulations encompass emollients, occlusives, and humectants, which can be classified into ointments, creams, oils, gels, or lotions. Although lotions and creams are generally more user‐friendly and less greasy than ointments, lotions possess a higher water content and tend to evaporate rapidly upon application, leading to a diminished overall contribution to skin hydration.^329^ Except for a novel category of prescription emollient devices, which includes specialized cream formulations containing various lipids and ingredients such as ceramides and filaggrin breakdown products, most moisturizers are available over‐the‐counter and do not require a prescription for use.^326^ Moisturization is the cornerstone of treatment for patients with mild AD and plays a crucial role in managing moderate to severe cases, and it is advisable to select moisturizers free from additives or fragrances, as these can further irritate inflamed skin.^390^ Phototherapy is an effective physical therapy option for patients with moderate to severe AD, particularly when other treatments prove insufficient. It is typically recommended as a second‐line or adjunctive treatment for both children and adults.^328^ Narrow‐spectrum UVB phototherapy is commonly used, as it penetrates the epidermis and reaches the upper dermis, where it is absorbed by cells, altering DNA expression and promoting the release of anti‐inflammatory cytokines.^391^, ^392^ Furthermore, psychological interventions, dietary management, and allergen avoidance can also contribute to improving patients' symptoms and overall quality of life.^393^, ^394^, ^395^ Additionally, these adjuvant therapies can be utilized in conjunction with other treatment modalities and complemented by healthy lifestyle habits to potentially impede the progression of AD.

## 5 CONCLUSION AND PROSPECTS

AD is a complex multifactorial disease, involving the interaction of genetic, immune, skin barrier, and environmental factors in its pathogenesis. Dysregulation of the immune response in AD is a complex issue that impacts antigen response, inflammation induction, skin structural integrity, and pruritus. Addressing this imbalance in immune response may offer significant benefits in the management and development of AD. The interactions among various skin components, including KCs, fibroblasts, and skin‐resident immune cells such as DC, intricately regulate the onset and progression of AD. Therefore, further research is essential to fully elucidate how these functionally distinct immune cells coordinate immune responses in AD.

Today, emerging therapeutic approaches offer a range of new options for managing AD, targeting different pathological mechanisms with demonstrated efficacy and safety. Treatments such as JAK inhibitors, biologics, PDE4 inhibitors, and microbial therapy not only fill gaps in traditional therapies but also have the potential to induce complete or nearly complete regression of skin lesions, significantly enhancing patients' quality of life and offering renewed hope for those with refractory AD. Despite the progress made in the treatment of AD in recent years, there are still many challenges. Emerging treatments for AD will also carry potential side effects, the possibility of developing treatment tolerance, and the risk of drug dependence. Furthermore, long‐term treatment necessitates that patients adhere to prescribed medication and care protocols. However, fluctuations in their condition and the side effects of medication often lead to fatigue and anxiety, which can adversely impact treatment effectiveness.

With advancements in technologies such as genomics, the future holds promise for patient stratification based on phenotypic characteristics and biomarkers. This will facilitate the identification of individuals with diverse disease trajectories and responses to targeted therapies, paving the way for personalized treatment. The individualized treatment of AD will become a future trend, providing patients with more precise and effective treatment plans. Moreover, the integration of digital health technology enables doctors to remotely monitor patients via mobile applications, facilitating improved management of their conditions. This approach has the potential to become a significant trend in the future. In addition, innovative therapies such as gene therapy and microRNA‐targeted therapies show promise for AD and warrant further exploration.

In summary, our review provides a comprehensive overview of underlying pathologies in AD, with a specific focus on immunological imbalances and cellular interactions, while also outlining the experimental models used for studying AD. Additionally, we extensively discuss therapeutic interventions in AD to provide guidance for investigating the mechanisms of AD and the clinical utilization of drugs, and anticipating to prevent the onset and progression of AD, as well as atopic complications.

## AUTHOR CONTRIBUTIONS

Chengcheng Yue and Jiong Li have conceptualized and designed this article. Chengcheng Yue, Hong Zhou, Xiaoyan Wang, Jiadong Yu, Yawen Hu, Pei Zhou, Fulei Zhao, Fanlian Zeng, Guolin Li, Ya Li, Yuting Feng, Xiaochi Sun, Shishi Huang, Mingxiang He, Wenling Wu, and Nongyu Huang have substantially contributed to the literature search and drafting of the article. All authors have approved the final version of the article and agreed on its integrity and intellectual content.

## CONFLICT OF INTEREST STATEMENT

The authors declare no conflict of interests.

## ETHICS STATEMENT

Not applicable

## Data Availability

Not applicable.
